# Non-toxic near-infrared light-emitting diodes

**DOI:** 10.1016/j.isci.2021.102545

**Published:** 2021-05-15

**Authors:** Kunping Guo, Marcello Righetto, Alessandro Minotto, Andrea Zampetti, Franco Cacialli

**Affiliations:** 1 Department of Physics and Astronomy and London Centre for Nanotechnology, University College London, London WC1E 6BT, UK

## Abstract

Harnessing cost-efficient printable semiconductor materials as near-infrared (NIR) emitters in light-emitting diodes (LEDs) is extremely attractive for sensing and diagnostics, telecommunications, and biomedical sciences. However, the most efficient NIR LEDs suitable for printable electronics rely on emissive materials containing precious transition metal ions (such as platinum), which have triggered concerns about their poor biocompatibility and sustainability. Here, we review and highlight the latest progress in NIR LEDs based on non-toxic and low-cost functional materials suitable for solution-processing deposition. Different approaches to achieve NIR emission from organic and hybrid materials are discussed, with particular focus on fluorescent and exciplex-forming host-guest systems, thermally activated delayed fluorescent molecules, aggregation-induced emission fluorophores, as well as lead-free perovskites. Alternative strategies leveraging photonic microcavity effects and surface plasmon resonances to enhance the emission of such materials in the NIR are also presented. Finally, an outlook for critical challenges and opportunities of non-toxic NIR LEDs is provided.

## Highlights

• Telecommunications and biomedical sensors will hugely benefit from the development of non-toxic NIR LEDs.
• Both organic and hybrid materials reported remarkable performance in the 700- to 1000-nm range.
• Aggregation and the energy gap law represent the toughest hurdles to overcome for efficient NIR emission in organic materials.
• Leveraging AIE, TADF, and hybrid materials have proven successful in improving efficiencies.

## Graphical abstract

**Figure:**
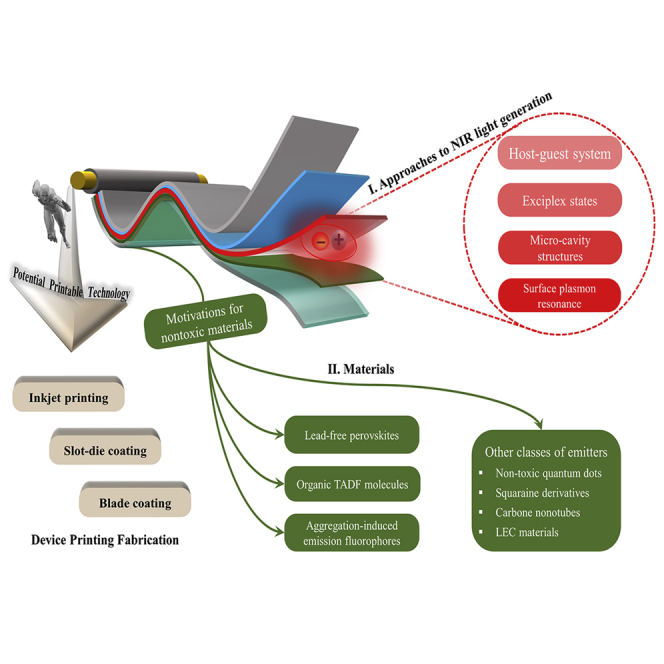

## 1 Introduction

Over the past years, optoelectronics underwent a paradigmatic shift of device form factors from fixed and rigid to more diverse form– flexible, bendable, and even stretchable architectures (Wang et al., 2019c; Ahn and Hong, 2014; Rogers et al., 2010). Flexible optoelectronics is paving the way to wearable devices, which carry a significant number of benefits including lightweight, high flexibility, and multiphoton signal generation/collection (Yang and Gao, 2019; Lochner et al., 2014). Using advanced printing techniques, flexible optoelectronics can be manufactured in mass production and large areas, up to hundreds of square meters (Khan et al., 2020), thereby redefining the optoelectronics scene toward fast and low-cost fabrication.

In bioelectronics in particular, research on light-emitting diodes (LEDs) has focused on topics such as electronic skin, biocompatible lasing, tattooable devices, and so on. (Vosgueritchian et al., 2013; Umar et al., 2019; Chen et al., 2018; Barsotti et al., 2021) Concurrently, next-generation printable optoelectronics are driving innovations toward so-called Internet-of-Things applications, which promise printable flexible sensors and actuators to be connected by networks, and thus enable interfacing of optoelectronic devices with “biology and nature” in addition to manufacts (Ray et al., 2019; Yang and Gao, 2019).

From a manufacturing point of view, the adoption of printed electronics is considered crucial in a variety of industries to improve the cost competitiveness against conventional display and solid-state lighting technologies (Arias et al., 2010). In fact, significant advantages of organic LEDs (OLEDs) compared with inorganic ones are afforded by their mechanical conformability for the integration with diverse substrates and inexpensive materials suitable for solution-processing deposition. More importantly, printed OLEDs could be more easily disposed of owing to their inherent non-toxic nature, thereby changing their life cycle from production to recycling (Han et al., 2017).

**Figure 1:**
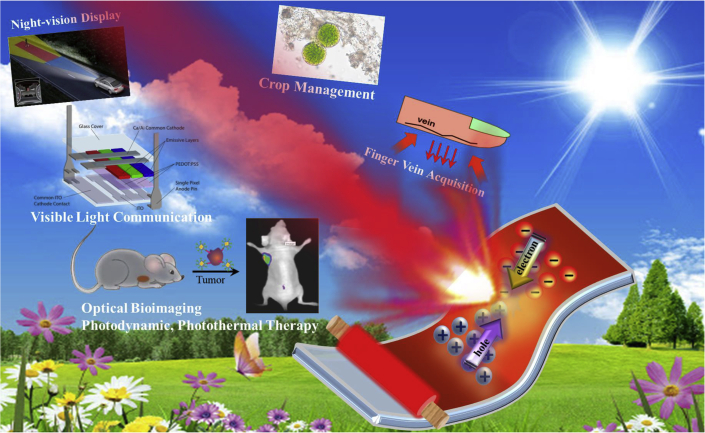
Schematic illustration for the application of NIR LEDs (Kühl et al., 2020; Haigh et al., 2015; Neuman et al., 2015; Veldhuis et al., 2020).

Envisioning these developments, environmental and economic concerns stimulate intense research for biocompatible, earth-abundant, and cost-efficient printable semiconductor materials. Given their chemical tunability, high quantum efficiency, and compatibility with solution-processing techniques, organic semiconductors have attracted widespread attention not only in the realm of academic research but also in industrial research and development (Rim et al., 2016; Kuehne and Gather, 2016). Interestingly, hybrid organic-inorganic perovskites, in particular lead halide perovskites (LHPs), are catalyzing further interest owing to their excellent optoelectronic performance. Although lead toxicity remains a significant concern, recent research studies indicate possible ways to achieve similar results with less-toxic or non-toxic perovskites (Zhuang et al., 2019). Notably, investigating the potential of organic and hybrid materials, such as near-infrared (NIR) emitters in printable LEDs, is tremendously important for a broad range of applications, as captivatingly illustrated in Figure 1, that is, starting from night-vision displays to enhance the visibility of car drivers (Kallhammer, 2006) to improving precision agriculture by enabling acquisition of accurate information on crop conditions (e.g. NIR illumination can be used to find optimal harvest times of crops by measuring crop ripeness) (Bogue, 2017). Furthermore, NIR radiation is virtually invisible to the human eye, and by expanding the available bandwidth, NIR LEDs are excellent candidates for integration as transmitters in visible light communication links, as recently reported by our group (Haigh et al., 2015; Minotto et al., 2020; Le et al., 2014) and others.

Interestingly, the first NIR window (NIR-I) that spans from 700 to 1000 nm is considered as the “optical transmission window” of biological tissues (Smith et al., 2009), where absorption and scattering of the excitation and emitted light, as well as autofluorescence, are reduced. For instance, larger light penetration in this range opens up the prospect of deep-tissue imaging with higher contrast (Kumar et al., 2009). For this reason, the NIR spectrum range is widely used for *in vivo* high-contrast optical imaging, targeting tumor cells (already proved in small animals), and even for photothermal therapy applications (Liu et al., 2018a; Sun et al., 2016; Neuman et al., 2015). In addition, NIR LEDs have also been used to capture human finger-vein-based personal identification to enable secure authentication (Veldhuis et al., 2020).

It is worth noting that the definition of the shorter wavelength end of the NIR in the literature is generally taken to be 700 nm, whereas the longer wavelength end is not clearly defined. In view of the fact that emission in the solid state at wavelengths beyond 1000 nm is rather weak for organic semiconductors, we will focus our attention in the following section on the NIR-I range 700–1000 nm and will refer to this range as the NIR.

Herein, we review the latest progress in the field of non-toxic organic and hybrid NIR LEDs, with a focus on solution-processed materials for potentially printable devices. This review starts by discussing the motivations for non-toxic materials (Section 2), we then recall different approaches to generate and promote NIR emission (Section 3), and summarize recent achievements in the development of highly efficient NIR LEDs, with special attention to lead-free perovskite materials, thermally activated delayed fluorescence (TADF) molecules, as well as aggregation-induced emission-active fluorophores (Section 4).

## 2 Motivations for non-toxic materials

With continued global growth and development, it is increasingly evident that finite energy resources are being depleted at an accelerated rate. The global energy demand has reached alarming levels and may eventually prove unsustainable. Many new technologies incorporating earth-abundant, affordable, and functional materials have been developed in the last few decades. These materials include metals, oxides, hybrid compounds, and the most commercial semiconductors, such as silicon or III–V semiconductors, owing to their high charge-carrier mobility, small exciton binding energy, and high stability (Yang and Ma, 2019). However, with the emergence of environmental issues, one of the main targets for prototype development is to match sustainability requirements, with a focus on materials that do not harm humans or the environment (Lee et al., 2017a).

The demand for advanced biodegradable or biocompatible materials and devices for a sustainable future is powerfully fueling the so-called “green electronics.” This is indeed increasing very rapidly, mainly as a result of the significant growth of electronic waste that has followed proliferation of electronic devices at an unprecedented pace (Liu et al., 2020b). For example, toxic elements such as gallium, antimony, or lead are widely exploited to manufacture highly efficient optoelectronic devices but raise environmental concerns over the device life cycle, from massive industrial production to subsequent hazardous disposal (Lim et al., 2011; Chao et al., 2018). To address these concerns, the European Union (EU) issued the “Restriction of Hazardous Substances” (RoHS) directive (European Commission, 2011). Nowadays, many other countries have also started to enact their own versions of an “RoHS” legislation, which also specifically regulate the manufacture, import, and distribution of electronics and electrical equipment within a country (Liu et al., 2020d). Before the RoHS directive, the EU also passed the Waste Electrical and Electronic Equipment Directive in 2002, which required improving the environmental performance of all operators involved in the life cycle of electrical and electronic equipment, for example, producers, distributors, and consumers and especially for those operators directly involved in the treatment of electronics waste (Menon et al., 2015). At the market scale level, even some minor measures needed at the end of the life cycle would have huge effects on the overall material cost. As this is a significant disincentive for large-scale adoption of devices based on toxic materials, there is huge interest in replacing these materials with less-toxic ones.

Nontoxic organic materials are an emerging paradigm in the field of carbon-based advanced functional materials. Current effort on these materials is aimed at achieving highly ambitious goals, including the integration of electronics into living tissues, with the aim of attaining biochemical monitoring and diagnostic and drug delivery tasks, without compromising the need to obtain human and environmentally benign solutions (Irimia-Vladu, 2014).

As testified by the case of active-matrix organic LED displays, for almost two decades, and thanks to the promise of their low-cost production and (in perspective) their potential sustainability, purely organic semiconductors have become the subject of intense industrial and academic investigation. By combining two of the most significant technological advances of the last century (i.e., semiconductors and plastics), these materials contributed to the inception of a wider area of conformable electronics applications. Crucially, organic materials with relevant optoelectronic properties are solution-processable and can be engineered to be biocompatible (i.e., suitable for integration into the “direct periphery” of the human body for leisure or healthcare applications) (Zimmermann et al., 2019).

Interestingly, however, organic-inorganic hybrid materials are also a promising alternative, which might have the potential to comply with RoHS limitations (such as <100 ppm Cd and <1000 ppm Pb contents). Recent efforts are being dedicated to exploitation of alternative materials containing non-toxic elements such as Sn, Ge, Bi in combination with organic compounds but excluding heavy metals. An alternative approach is that of encapsulating potentially toxic compounds “hermetically” within a non-toxic shell (Wang et al., 2011, 2019b; Uoyama et al., 2012). While this field has demonstrated remarkable progress, the performance of the devices incorporating such materials is still not comparable with that of devices making use of (heavy) metal-containing semiconductors, such as Cd- or Pb-based ones, thereby calling for further efforts.

## 3 Approaches to NIR light generation

### 3.1 The quest for efficient NIR LEDs

Despite some differences between purely organic and organic-inorganic hybrid semiconductors, LEDs based on these materials share similar operational principles. Charge-transporting and electroluminescent semiconductor materials are typically sandwiched between two electrodes, one of which is semitransparent to allow for effective light extraction. When a “forward” operating voltage is applied to the electrodes, charge carriers can be injected into the semiconductor layers, where they combine to form an excited state (with sizable binding energy and termed an “exciton”) that can then decay to the ground state. Such excited states are essentially the same as those produced by optical excitation, and therefore, as in the case of the photoluminescence, the competition between radiative and nonradiative of processes eventually determines the emission efficiency per exciton produced.

The external quantum efficiency (EQE) in the case of electroluminescence (EL) can be written as:

(Equation 1) $$EQE=\xi \cdot IQE=\xi \cdot \gamma \left[r_{st}\cdot \eta_{FL}+\left(1-r_{st}\right)\cdot \eta_{PH}\right]$$

where *r*_*st*_ is the singlet to total number of excitons ratio, *η*_*FL*_ (*η*_*PH*_)is the fluorescence (phosphorescence) efficiency of the emitting layer, and *γ* is a factor that takes into account the probability of mutual capture of positive and negative carriers and that is thus related to their populations imbalance (more precisely the maximum value of gamma is the ratio of minority to majority carrier populations).

The light outcoupling efficiency *ξ* and the population balance factor *γ* depend strongly on the LED architecture, but *γ* is also controlled by materials purity/structure, such as traps in charge transport layers (CTL), CTL/emissive layer (EML) interfaces, and/or the nature of the EML. Improvements of the singlet–to–the total number of excitons ratio *r*_*st*_ and the fluorescence and/or phosphorescence quantum efficiency usually involve development of the active materials (Park et al., 2019), which are the main subject of this review. Clearly, one needs to develop both the LED architecture and the advanced materials to obtain “the best of all possible worlds.”

Within this framework, intensive efforts have been devoted and are still going on to replace the most efficient NIR LEDs, which make use of organometallic phosphorescent complexes or Pb-based halide perovskites, with metal-free fluorescent polymers, small molecules, and lead-free halide perovskites.

All device investigations and results obtained so far can be accounted for with a singlet-to-triplet formation ratio as expected as per “simple spin statistics,” that is with *r*_*st*_ = 0.25. We also note that TADF materials (described in detail in Section 4.2) achieve an “effective” *r*_*st*_ > 0.25, thanks to thermally assisted promotion of triplets to singlets. For these reasons, optimization of the photoluminescence efficiency (*η*_*PL*_) is crucial to achieve high EQEs.

Achieving efficient light emission is also more challenging in the NIR compared with the visible because of the need to address the problems connected with unfavorable aggregation patterns and the so-called “energy gap law (EGL).” Turning to aggregation first, we note that for organic materials, NIR emission is generally obtained by synthesizing extended conjugated π-frameworks that are thus intrinsically prone to cofacial aggregation. Resulting H-aggregates (or related ones with significant H-character) (Oleson et al., 2019) display an at least partially (dipole-)forbidden lowest-energy transition, therefore characterized by lower radiative rates (*k*_*r*_) compared with those of the isolated molecules (Varghese and Das, 2011; Hestand and Spano, 2018). Thus, approaches such as chromophore dilution in “solid” solutions by blending the emitter in a host (Section 3.2) and material development targeting aggregation-induced emission (AIE) (Section 4.3) have been developed to either avoid altogether or “to beneficially engineer” the process of aggregation.

On the other hand, suppressing nonradiative recombination processes (and thus the nonradiative rate *k*_*nr*_) implies addressing or bypassing the “EGL.” The EGL “prediction” is the key result of a theory of radiationless transitions developed by Englman and Jortner (Siebrand, 1967; Englman and Jortner, 1970). In the case of low temperatures and in the so-called “weak vibrational coupling” regime, that is, Δ*E*/(*S*_*M*_*ℏω*_*M*_)≫1, with Δ*E* the energy of the optical transition, $S_{M}=\frac{1}{2}{\text{Δ}}_{M}^{2}$, a measure of the "distortion" of *ω*_*M*_in the excited state (and where, in turn, *ω*_*M*_ = 2*πν*_*M*_ denotes the maximum angular frequency of the acceptor vibration in the ground state and Δ_*M*_ is the fractional displacement in vibration *ω*_*M*_between the thermally equilibrated ground and excited states), the theory predicts that the nonradiative recombination rate is exponentially dependent on the ratio between Δ*E* and that of high-frequency molecular vibrations (*ℏω*_*M*_) through a “molecular” constant *K* as:

(Equation 2) $$k_{nr}\;\propto \exp\left(-K\frac{\text{Δ}E}{\hbar \omega_{M}}\right)$$

with

(Equation 3) $$K=\ln\left(\text{Δ}E/\lambda_{m}\right)-1$$

where *λ*_*m*_ is the reorganization energy that is a molecular parameter describing the degree of molecular distortion associated with an electronic transition. Clearly, excitations in the NIR (∼10− 14 × 10^3^ *cm*^−1^) can be dissipated (relatively) easily by molecular acceptor modes reaching up to 3000 wavenumbers (∼0.37 eV). The law has been experimentally confirmed for series of chemically similar materials (Caspar and Meyer, 1983), although the dependence of *K* on molecular structure and properties makes it difficult to make absolute predictions when comparing molecules with significantly different chemical and therefore vibrational structure. Together with the tendency to form poorly emissive aggregates for large conjugated systems, the EGL constitutes the major factor limiting the quantum efficiency of NIR OLEDs.

Interestingly, however, the aforementioned Equation (2) also suggests leveraging control of the constant *K* to bypass the detrimental action of the EGL. In other terms, because the EGL is brought about by the coupling of electronic and vibrational states (even in the weak coupling regime), the decoupling of electronic excitations and molecular vibrations, as observed for example in the case of exciplexes (Section 3.2), should provide a way for bypassing the EGL. Although the EGL has also been observed in exciplex-forming donor-acceptor (D-A) pairs (Ullbrich et al., 2019; Gould and Farid, 2007), a judicious “supramolecular engineering” of *K* was successfully proposed recently by Wei et al to mitigate the EGL (Wei et al., 2020). Namely, in the latter work, it was proposed that for molecular aggregates in the strong excitonic coupling regime, the monomeric reorganization energy is partitioned (i.e., each monomer shares only a fraction of the *λ*_*m*_, as a result of the reduced vibrational coupling). Hence, the effective reorganization energy *λ*_*eff*_ diminishes with increasing exciton delocalization lengths (over N molecules), *λ*_*eff*_ = *λ*_*m*_/*N*. Specifically, they designed Pt(II) complex aggregates emitting at wavelengths >800 nm and with an exciton delocalization length over a number of molecules N ∼ 5–9, which afforded photoluminescence quantum yield (PLQY) values up to 12%. Such Pt (II)-complex-based NIR LED achieve a remarkable and unprecedented combination of EQE (up to 2.14%), radiance (41.6 W sr^−1^ m^−2^) and, most importantly, an emission (peak) wavelength of 930 nm.

A synergistic approach to address the limitations imposed by intersystem crossing (ISC), the EGL, and aggregation quenching while simultaneously boosting the radiative rate by increasing the emitters oscillator strength, and restricting the investigation to heavy-metal-free materials is also possible, as we have recently shown in collaboration with the synthetic group led by H.L. Anderson at Oxford. Here, the use of porphyrin oligomers with increasing length as emitters is found to attenuate the effects of the EGL by suppressing the nonradiative rate growth and to increase the radiative rate via enhancement of the oscillator strength while bulky side chains simultaneously suppress aggregation quenching. Interestingly, we found that the logarithmic rate of variation of the nonradiative rate versus the energy gap was suppressed by an order of magnitude with respect to previous studies. Organic LEDs with a maximum EQE of 3.8% (average ∼1.1%) at 850 nm were also demonstrated (Minotto et al., 2021). Crucially, the presence of conjugated triple-bond-based bridges between the porphyrins allows effective intramolecular electronic coupling among the macrocycles and thus enables the singlet exciton to delocalize over increasing portions of the molecule, thereby forcing an increasing mismatch of the spatial extent of the singlet and of the triplet excitons in view of the intrinsically localized nature of the triplets. Such a mismatch is expected to suppress ISC and therefore the nonradiative rate (*k*_*nr*_). In addition, exciton delocalization is also expected to favor decoupling from vibrational ladders, as beautifully argued by Wei et al. These results provide a general strategy for designing high-luminance NIR emitters.

If we turn instead to inorganic and hybrid NIR emitters, the effects of the EGL are less important owing to the lower energy of the relevant phonons (e.g., optical phonon frequencies in LHPs are generally lower than 150 cm^−1^) (Sendner et al., 2016). However, further limitations emerge as trap-mediated recombination dominates over nonradiative processes, as in conventional semiconductors (Das et al., 2020; Righetto et al., 2017). Differently from organic materials, for which the primary excitations are excitonic in nature (i.e., characterized by binding energies much greater than kT for the hole-electron pairs, typically several hundred meV), perovskites are characterized by comparatively low exciton binding energies (<50 meV), stemming from combined greater dielectric constants (*ε*_*r*_∼10) and low carrier effective masses ($m_{e}^{*},m_{h}^{*}∼0.1\;m_{0}$, where *m*_0_ is the electron rest mass) (Yang et al., 2017; Miyata et al., 2015), also associated with lower radiative rates. The “nearly free-carrier” nature of the excitations also favors efficient quenching at “defects,” such as lead vacancies (V_Pb_) and interstitial halides (X_i_) (Mosconi et al., 2016). Interestingly, however, the large majority of trap states have a “shallow character” (Righetto et al., 2020b). Therefore, a “suitable” passivation of residual deep traps can help maintaining the associated nonradiative rate at an acceptably low level (*k*_*trap*_ ∼ 10^5^ *s*^−1^) (Sum et al., 2020). As the defect tolerance is related to the electronic properties of lead, non-toxic perovskites should use elements with a similar electronic structure (as further discussed in Section 4.1).

As demonstrated by Cho et al. (Cho et al., 2015), reducing grain sizes is a viable strategy to induce a stronger spatial confinement, thereby reducing dissociation and enhancing radiative recombination. Accordingly, higher luminescence efficiency was demonstrated for perovskite nanocrystal (NC) thin films (Sum et al., 2020). Therefore, lower dimensional non-toxic perovskites (e.g., 2-dimensional [2D] and NCs) are also emerging as promising efficient NIR emitters, and we expect future work to focus on their implementation in LED devices (Vargas et al., 2020).

### 3.2 Host-guest system

As per Equation (1), efficient LEDs need balanced charge transport as well as a high conversion efficiency of excitons to light. Host-guest organic semiconductor systems emerged as a crucial strategy to decouple these two processes and to fabricate highly efficient devices (Tao et al., 2011; Cacialli and Stoneham, 2002). In this strategy, the EMLs comprise a highly emissive guest (e.g., a small molecule, a metallic complex, or a polymer) blended and diluted within a host matrix, which mediates the carrier/exciton transport. Although the use of these blends introduces greater complexity, which involves chemical and thermodynamics concepts (Cacialli and Stoneham, 2002), they also carry two main advantages. First, the dilution of the guest helps to preserve its emissive properties from concentration quenching effects related to aggregation phenomena, as described previously. Second, depending on the specific band alignment and transport properties of the host matrix, excitations can be transferred from the host to the guest, thus effectively decoupling transport and emission. For instance, singlet excitons can be formed in the host under electrical excitation and then transferred to the guest via Förster energy transfer. Alternatively, electrons and holes can be transported by the host and directly recombine on the guest. In the case of phosphorescent guests, triplet excitons can also be harvested via Dexter energy transfer, thereby paving the way to nearly unitary internal quantum efficiencies (Tao et al., 2011). Crucially, although fluorescent materials do not harvest triplet excitons and thus their maximum internal quantum efficiency is limited to 25%, fluorescent molecules are typically “heavy-metal-free” and therefore more environmentally sustainable compared with phosphorescent heavy-metal complexes.

Guest molecules having a smaller energy gap are generally dispersed within an organic matrix with a wider gap. The highest occupied molecular orbital (HOMO) and the lowest unoccupied molecular orbital (LUMO) levels of the commonly used host materials for NIR emitters are summarized in Table 1, almost all host materials exhibit a larger HOMO-LUMO separation with respect to the guest molecules. Notably, the fine tuning of this alignment plays a fundamental role in tuning the carrier transport (Sanderson et al., 2019).

**Table 1:** Photoluminescence and electroluminescence characteristics of recent host-guest systems for NIR emission.

Class	Host-guest EML	Host			λ_EL_ [nm]	EQE_max_ [%]	Ref.
		λ_PL_ [nm]	HOMO [eV]	LUMO [eV]			
Small molecule	CBP: 30% TBtz1	400	5.8	2.7	702^a)^	1.52	(Sudyoadsuk et al., 2020)
	CBP: 30% TBtz2	400	5.8	2.7	723^a)^	1.22	(Sudyoadsuk et al., 2020)
	CBP: 10% CAT-1	390	5.6	2.4	719^b)^	1.6	(Congrave et al., 2019)
	mCP: 20% Nd(TTA)_3_phen	395	5.9	2.4	890^b)^	0.02	(Shahalizad et al., 2017)
	TPBi: 30% TPA-QCN	381	6.2	2.7	700^b)^	9.4	(Li et al., 2017)
	TCTA: APDC-tPh (1:1)	385	5.8	2.4	730^b)^	0.1	(Hu et al., 2020)
	Alq_3_: 40% DTPS-PT	512	5.6	2.8	842^b)^	NA	(Jiang et al., 2019)
	ADO-TPA: 20% APDC-DTPA	645	5.4	3.2	735^b)^	2.7	(Hu et al., 2018a)
	F8BT: 2.5% l-P6(THS)	770	5.1	3.5	850^a)^	3.8	(Minotto et al., 2021)
Polymer	PVK: 20% 2TPA-PPDC	450	5.8	2.2	696^a)^	0.59	(Choulis et al., 2005; Zhang et al., 2019)
	F8BT: 0.5% NIRBDTE	550	6.0	3.3	720^a)^	1.1	(Zampetti et al., 2017)
	MEH-PPV: 5.0% DD	590	5.1	2.7	720^a)^	0.3	(Ostrowski et al., 2003)
	PIDT-2TPD: 0.5% BTT	700	6.1	3.7	840^a)^	1.15	(Minotto et al., 2018)

**Figure 2:**
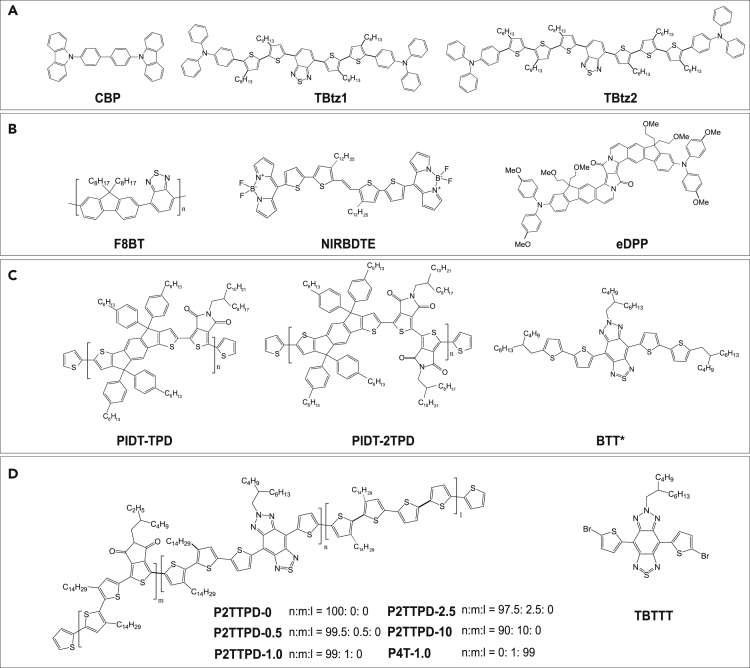
Molecular structures of small molecules and polymers used in host: guest NIR LEDs (A) Molecular structures of the CBP, TBtz1, and TBtz2 (Sudyoadsuk et al., 2020). (B) Molecular structures of the F8BT host polymer, NIRBDTE, and eDPP (Zampetti et al., 2017; Minotto et al., 2020). (C) Molecular structures of the PIDT-TPD/PIDT-2TPD host polymers and BTT∗ NIR dye (Minotto et al., 2018). (D) Molecular structures of the polymers P2TTPD-0, P2TTPD-0.5, P2TTPD-1.0, P2TTPD-2.5, P2TTPD-10, P4T-1.0, and guest TBTTT (Murto et al., 2016).

The devices were fabricated by ^a)^ solution-process; ^b)^ thermal evaporation. CBP = 4,4′-bis(N-carbazolyl)-1,1′-biphenyl, TBtz1-2 see Figure 2A, CAT-1 = see Figure 8D, mCP = 1,3-bis(9-carbazolyl)benzene, Nd(TTA)_3_phen is a Neodimium thenoyltrifluoroacetone and 1,10-phenanthroline complex, TPBi = 1,3,5-tris(1-phenyl- 1*H*-benzimidazol-2-yl)benzene, TPA-QCN = triphenylamine-quinoxaline-6,7-dicarbonitrile, TCTA = 4,4′,4″-tris(carbazol-9-yl)triphenylamine, ADPC-tPh = 3-([1,1″:3′,1″-terphenyl]-5′-yl)acenaphtho[1,2-b]pyrazine-8,9-dicarbonitrile, Alq_3_ = tris(8-hydroxyquinoline)aluminum(III), DTPS-PT = 5,5′-([1,2,5]thiadiazolo[3,4-c]pyridine-4,7-diyl)bis(N,N-diphenylthiophen-2-amine), APDC-TPA = 3,4-bis(4-(diphenylamino)phenyl)acenaphtho[1,2-b]pyrazine-8,9-dicarbonitrile, ADO-TPA = 5-(4-(diphenylamino)phenyl)acenaphthylene-1,2-dione, F8BT = poly(9,9′-dioctyl-fluorene-alt- benzothiadiazole), l-P6(THS) = linear meso-butadiyne-linked zinc porphyrin hexamer, PVK = Poly(9-vinylcarbazole), 2TPA-PPDC = 4-tert-butyl-N-(4-tert-butylphenyl)-N-phenylbenzenamine (TPA) and pyrazino[2,3-f][1,10]phenanthroline-2,3-dicarbonitrile donor acceptor system, NIRBDTE see Figure 2B, MEH-PPV = poly-[2-methoxy-5-(2-ethylhexyloxy)-1,4-phenylene] vinylene, PIDT-2TPD see Figure 2C, BTT = triazolobenzothiadiazole, DD = bis[(5,5′-10,20-bis[3,5-bis(3,3-dimethyl-1-butyloxy)-phenyl]porphinato)zinc(II)]ethyne.

Currently, in the vast majority of cases, the small-molecule OLEDs typically use common host materials as matrices (especially for TADF devices). Table 1 summarizes some representative host-guest system used in the fabrication of NIR OLEDs. For small-molecule host-guest systems, the most common host matrices mainly include 4,4′-bis(*N*-carbazolyl)-1,1′-biphenyl (CBP), 1,3-bis(9-carbazolyl)benzene (mCP), 1,3,5-tris(1-phenyl-1*H*-benzimidazol-2-yl)benzene (TPBi), 4,4′,4″-tris(carbazol-9-yl)triphenylamine (TCTA), tris(8-hydroxyquinoline)aluminum(III) (Alq_3_), and so on. The preference for these host materials derive from their availability, low cost, and well-documented photophysical properties, with countless examples of integration in high-performance visible OLEDs; in most cases, these materials are thermally evaporated although, interestingly, Sudyoadsuk et al. recently demonstrated solution-processed NIR OLEDs with CBP as the host in the active layer (Sudyoadsuk et al., 2020). All reported devices showed emission in the NIR region with an EL emission peaked at wavelengths longer than 700 nm, and the TBtz1-2 (Figure 2A) doped in CBP host exhibited much better performance than the nondoped devices, with maximum EQE (EQE_max_) of 1.22–1.52% for the doped devices and 0.26–0.48% for the non-doped devices.

Compared with small-molecular-weight host materials, polymers host matrices offer the specific advantage of solution processability. More importantly, thin films of polymeric materials are generally very smooth and uniform, enabling a greater control over film structure and morphology (Usta and Facchetti, 2015a). Furthermore, the possibility to tune effectively the solution rheological properties is advantageous for industrial printing processes, which require precise control over the whole films/device preparation cycle. Indeed, the fabrication of multilayers using solution deposition processes requires stacked layer to be insoluble toward solvents and processing temperatures involved in the fabrication of subsequent layers. The reduced solubility of polymers and their large bulk viscosity achieved by ad hoc chemical design increase the material choices especially when it is crutical to find materials soluble in orthogonal solvents for multilayered deposition via solution-processing techniques. Furthermore, polymers do not vaporize before decomposition, thus they are not susceptible to interlayer diffusion during the typical thermal cycles during device fabrication and typically exhibit robust mechanical properties, making polymer LEDs (PLEDs) potentially compatible with roll-to-roll fabrication on flexible substrates (Usta and Facchetti, 2015b).

It is commonly accepted that concentration quenching occurs in a host-guest system owing to molecular aggregation phenomena (Lee et al., 2017b; Pan et al., 2020; Kawamura et al., 2006; Bera et al., 2005). With increasing guest concentrations, the quantum efficiency generally drops and a bathochromic shift in the emission spectrum, indicative of an aggregate state, can be observed (Divayana and Sun, 2007). As an example, we draw attention to an acceptor-donor-acceptor-type NIR dye, referred to as NIRBDTE (Figure 2B), which was incorporated into poly(9,9′-dioctyl-fluorene-alt-benzothiadiazole) (F8BT) host for LEDs. With increasing NIRBDTE concentrations from 0.5 to 5 wt%, a progressive red shift from 720 to 800 nm was observed in doped PLEDs. Notably, the lowest concentrated NIRBDTE systems exhibited maximum EQEs up to 1.1% (Zampetti et al., 2017).

For the small-molecule-doped PLEDs, the effective strategy requires optimization of the doping concentration to reach the best compromise between the opposite needs of suppressing concentration-caused emission quenching and exciton annihilation and of ensuring efficient energy transfer from the host to quench its luminescence and afford purely NIR emission. Very recently, we demonstrated new far-red/NIR LEDs with a 650 to 800 nm emission range by incorporating a fluorescent π-expanded diketopyrrolopyrrole dye (eDPP) blended in an F8BT polymer matrix (Minotto et al., 2020). In this case, an optimal dopant concentration of 0.2 wt% was found to minimize aggregation quenching, while still affording good spectral purity. These LEDs showed EL peaking at 670 nm, with ∼50% of photons falling in the NIR spectral range (here defined as λ > 700 nm), radiances >3 mW/cm^2^, and EQEs reaching 2.72%. In another example, our group also reported a novel red/NIR emitting polymeric host (PIDT-2TPD), specifically tailored to enhance charge transport and spectral overlap, and a modified triazolobenzothiadiazole (BTT∗) emitter, as shown in Figure 2C. PLEDs incorporating PIDT-2TPD: 0.5% BTT∗ blends exhibited virtually pure NIR EL emission (≈98% in the NIR region) peaked at 840 nm with a turn-on voltage of only 1.7 V, EQE up to 1.15% (Minotto et al., 2018). Notably, polymer PIDT-2TPD exhibits significantly improved characteristics compared with previously reported PIDT-TPD matrices.

Despite these very promising achievements obtained with polymer hosts, the development of small-molecule-doped PLEDs is often still lagging behind all-small-molecule OLEDs. For instance, a new host material (ADO-TPA) with bipolar transporting characteristics was designed for NIR OLED, achieving 2.7% EQE at 735 nm (Hu et al., 2018a). Novel TADF molecule, a boron difluoride curcuminoid derivative, doped in the common small-molecule CBP host enables a maximum EQE close to 10% at 721 nm (Kim et al., 2018).

An alternative approach is to copolymerize the NIR moiety with a polymer host with a wider bandgap (Tregnago et al., 2015) so as to restrict the chances of the chromophores to aggregate freely. For example, the successful use of the wide bandgap poly[3,3′-ditetradecyl-2,2′-bithiophene-5,5′-diyl-*alt*-5-(2-ethylhexyl)-4*H*-thieno[3,4-*c*]pyrrole-4,6(5*H*)-dione-1,3-diyl] (P2TTPD) as a host (related chemical structures are shown in Figure 2D) incorporating the narrow gap 6-(2-butyloctyl)-4,8-di(thiophen-2-yl)-[1,2,3]triazolo[4′,5′:4,5]benzo[1,2-*c*]-[1,2,5]thiadiazole (TBTTT) molecule as the NIR emitter was reported recently (Murto et al., 2016). Pure NIR emission peaking around 900 nm was obtained from PLEDs based on P2TTPD host, thanks to efficient energy and charge transfer and exciton formation at the TBTTT sites. Such a strategy provides an encouraging and promising insight for development of this branch in NIR PLEDs.

### 3.3 Exciplex systems

**Figure 3:**
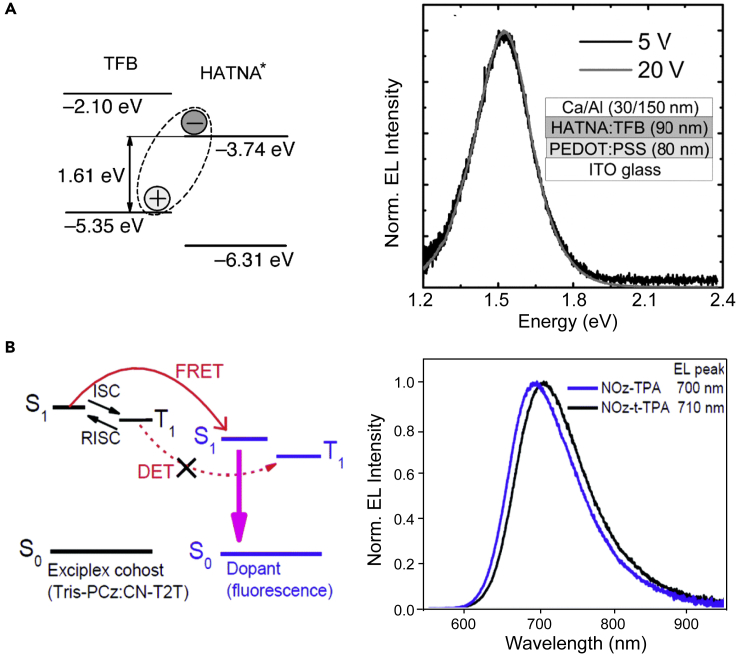
Exciplexes for NIR electroluminescence (A) Energy levels of the TFB and HATNA and corresponding EL spectra of LED taken at 5 and 20 V. The inset shows the LED structure. Reproduced and adapted from Appl. Phys. Lett. 105, 143304 (2014) with permission of AIP Publishing (Tregnago et al., 2014). (B) Schematic of the emission mechanism in the exciplex co-host system doped with fluorophores and corresponding EL spectra of LEDs. Reproduced and adapted with permission of the Royal Society of Chemistry, from J. Mater. Chem. C, 2020,8, 5704-5714 (Huang et al., 2020).

In some cases, the accumulation of electron and holes at the interface of hole and electron transport materials with large energy level offsets in an OLED leads to the formation of an “excited complex” or exciplex. These states have been widely exploited in monochromatic visible and hybrid white OLEDs, with improved device performance in some cases (Seino et al., 2014; Liu et al., 2015, 2016). Notably, this approach based on exciplex formation at a type II heterojunction (i.e., the lineup of the two semiconductor bandgaps has a staggered shape) might offer an additional pathway to circumvent the EGL (Section 3.1), by avoiding “crossing” of the energy surfaces of excited and ground states. NIR LEDs from exciplexes at polyfluorene/hexaazatrinaphthylene bulk heterojunctions were reported for example in 2014 (Tregnago et al., 2014). As shown in Figure 3A, the polymer-based LED shows EL peaking at 816 nm (1.52 eV, >98% of photons falling in the NIR). The NIR emission is achieved thanks to the large spectral shift of ∼1 eV from exciton to exciplex states.

More recently, exciplex-forming hosts have also been proposed to achieve barrier-free charge injection, unimpeded charge transport, and more generally as a cost-effective method as exciplexes can be simply formed by physical blending (Zhang and Xie, 2019). Huang et al demonstrated efficient NIR EL by exploiting the energy transfer between the exciplex-forming host blend and the fluorescent guest (Huang et al., 2020). As shown in Figure 3B, two chromophores have been applied as guest emitters to investigate the Tris-PCz:CN-T2T (1:1 in molar ratio) exciplex-forming host, with the latter acting as the energy donor. A thorough time-resolved characterization revealed significant differences in the energy transfer pathways, that is, Förster versus Dexter-type energy transfer between the exciplex-forming host and the fluorescent guest. NOz-*t*-TPA-doped device displayed an NIR emission peak at 710 nm with an EQE of 6.6%, which is among the highest values reported for metal-free NIR OLEDs around 710 nm. Nevertheless, such an efficient NIR device was fabricated by vacuum evaporation, which raises concerns about high production costs (discussed in detail in the following Section 5).

If we restrict ourselves to solution-processed NIR LEDs incorporating functional materials free from heavy or toxic metals, we are not aware of any further report of the utilization of exciplex states, thereby suggesting that exciplex systems for NIR LEDs are still largely unexplored. However, some intrinsic limitations arising from the presence of the local molecular interactions and low-charge transporting capability of the bulk heterojunctions derived from their intrinsic incompatibility may have caused a limited use of exciplexes for NIR light emission insofar.

### 3.4 Microcavity structures

Microcavity structures are a viable strategy to improve the color gamut, tailor the emission, and enhance light extraction from visible OLEDs (Lee et al., 2018; Thomschke et al., 2012; Chien et al., 2016; Grüner et al., 1996a). In traditional Fabry-Pérot microcavity architectures, the EML is generally sandwiched between a reflective electrode and a semitransparent electrode (Park et al., 2014). Such a structure applies to all OLEDs, while the semitransparent electrode mostly uses either a distributed Bragg reflector or a thin metal electrode (Wang et al., 2020a; Schwab et al., 2013).

**Figure 4:**
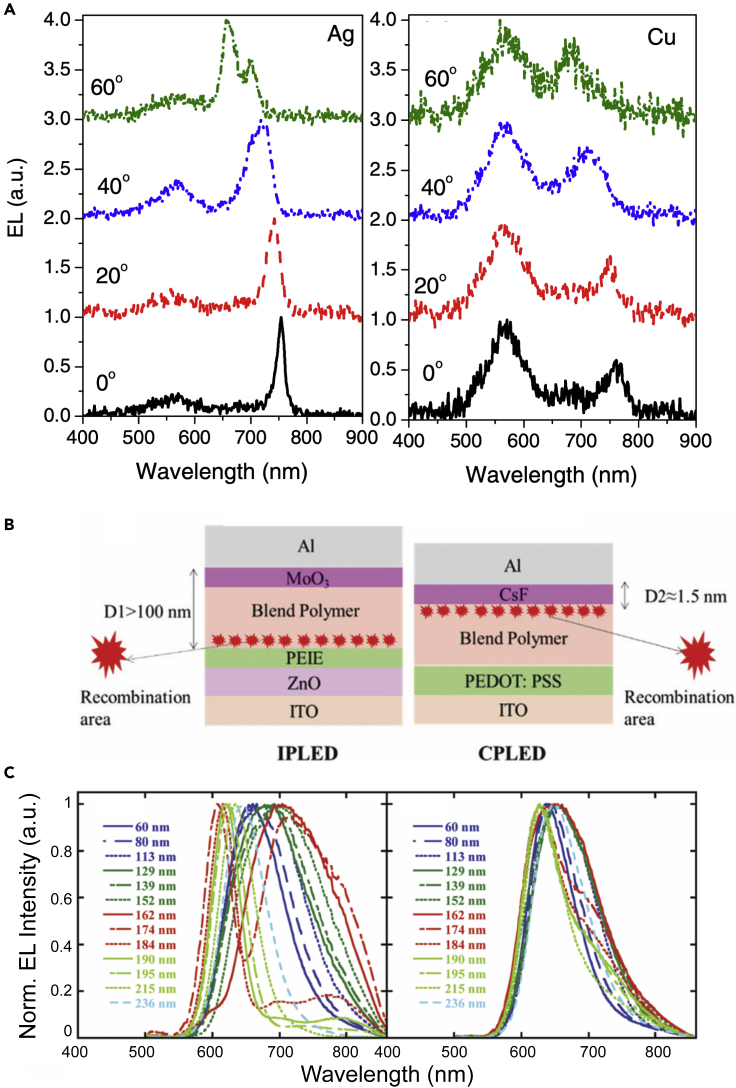
Leveraging microcavity effects for NIR electroluminescence (A) The EL spectra for microcavity OLEDs with different bottom mirror: (Left) Ag ~ 80 nm and (right) Cu ~ 80 nm. Reproduced and adapted with permission from Chem. Phys. Lett. 399 (2004) 446–450. Copyright 2004, Elsevier (Djurišić et al., 2004). (B and C) (B) Architecture and relative recombination area and (C) EL spectra based on different thicknesses of the emissive layer for an inverted polymer LED (IPLED, left) and a conventional polymer LED (CPLED, right). Reproduced and adapted with permission of the Royal Society of Chemistry, from J. Mater. Chem. C, 2019,7, 12,114-12,120 (Xu et al., 2019a).

Surprisingly, given the typically green emission of Alq_3_, Djurišić et al. reported the fabrication and characterization of NIR-emitting microcavity OLEDs incorporating NPB and Alq_3_ via vacuum evaporation (Djurišić et al., 2004). The authors concluded that triplet states of Alq_3_ are not involved owing to relatively short characteristic decay times (11–13 ns) and that the NIR emission arises through the interplay of the cavity resonance and the Alq_3_ film tail states. Two nontransparent copper (Cu) and silver (Ag) were used as bottom mirrors instead of semitransparent electrode, while Ag was used as cathode/top mirror. As shown in Figure 4A, it can be clearly observed that the dominant EL peak is located at ∼750 nm at normal incidence, and a blue shift with increasing viewing angle is observed as usual for Fabry-Pérot cavities (Grüner et al., 1996b). Moreover, the observed peak splitting of the NIR emission at larger viewing angles was attributed to the polarization mode splitting. Conversely, the device using the lossier Cu mirror presented a dominant emission at ∼570 nm, accompanied by a weak peak at ∼760 nm. This further demonstrated the thin Ag layer is a good candidate as an electrode thanks to its high reflectivity, also in agreement with previous literature (Grüner et al., 1996a).

Recently, Xu et al fabricated normal and inverted PLEDs featuring poly[2-methoxy-5-(2-ethylhexyloxy)-1,4-phenylene- vinylene] (MEH-PPV) blended with a red fluorescent material (PF-FSO10:PPF-FSO15-DHTBT10) as an active layer (Figure 4B). By tuning the thickness of the EML, the EL spectra of normal PLED devices remained almost the same, with only slight narrowing and broadening for the different thicknesses of the EML (Xu et al., 2019a). As shown in Figure 4C, the inverted PLED device yielded NIR emission with the main peaks located at 700 and 706 nm, with an EQE_max_ of 0.54 and 1.03%, respectively.

### 3.5 Plasmonic nanostructure-boosted NIR

**Figure 5:**
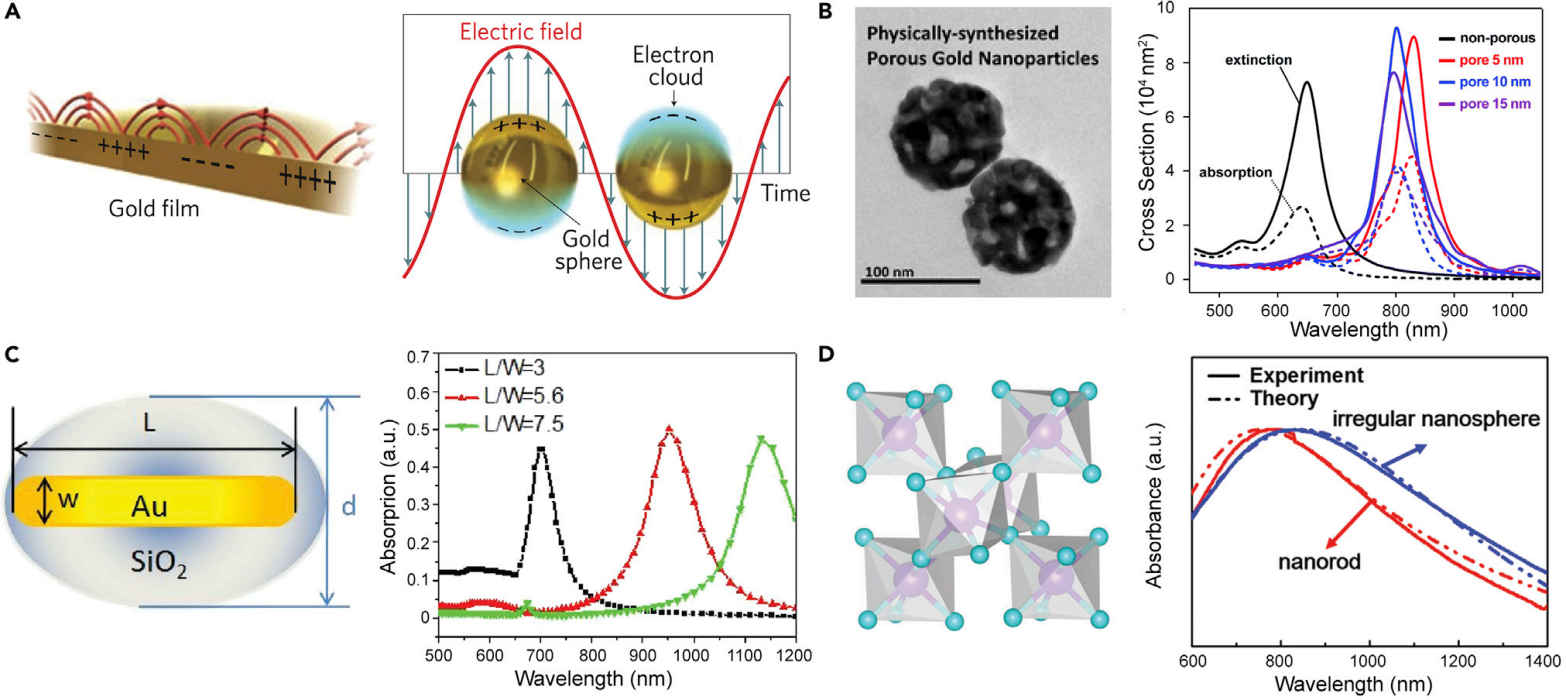
Plasmonic nanostructures for plasmon-enhanced NIR emission (A) Schematics for propagating surface plasmon polaritons along the dielectric-metal interface (left) and localized surface plasmons on the surface of metallic nanoparticles (right) Reproduced and adapted with permission from Nat. Photon. 5, 349–356 (2011). Copyright 2011, Springer Nature (Juan et al., 2011). (B) TEM images of porous gold nanoparticles and calculated extinction (solid) and absorption (dotted) cross-sections of non-porous gold particles and nanoporous gold particles modeled with pore diameters of 5, 10, and 15 nm. Reproduced and adapted with permission of the Royal Society of Chemistry, from Nanoscale, 2016,8, 15,514-15,520 (Park et al., 2016). (C) Schematic diagram of SiO_2_@AuNRs model and corresponding theoretical absorption curves of an individual SiO_2_@AuNR with a constant SiO_2_ thickness and varying aspect ratio. Reproduced and adapted with permission from Adv. Optical Mater. 2016, 4, 763–771. Copyright 2016, John Wiley & Sons (Luo et al., 2016). (D) Crystal structure of monoclinic MoO_2_, and the experimental and theoretical absorbance spectra of MoO_2_ nanocrystals with nanosphere and nanorod shapes. Reproduced and adapted with permission of the Royal Society of Chemistry, from J. Mater. Chem. B, 2017,5, 7393-7402 (Dou et al., 2017).

Subwavelength metallic nanostructures capable of supporting so-called localized surface plasmon resonances (LSPRs) are a highly effective route to augment emission of light from organic and polymer LEDs. An optimized coupling between organic semiconductors and these plasmonic nanostructures can bring about improved light extraction, decreased nonradiative decay, and increased luminescent efficiency (Lozano et al., 2013; Kochuveedu and Kim, 2014). These structures typically use particles or nanovoids to realize confined electron plasmas that can couple to the electromagnetic radiation, unlike so-called surface plasmon polaritons (SPPs) that although propagating along a (typically flat) metal-dielectric interface (in the x and y directions), decay in the z direction evanescently and therefore cannot be directly coupled to propagating light as illustrated in Figure 5A, unless *ad hoc* strategies or architectures are adopted (Juan et al., 2011). The higher (lateral) spatial confinement, the higher local field enhancements, and the better coupling with the radiative field make LSPR favorable over SPP for enhancing LEDs efficiencies. Moreover, as a consequence of the stronger confinement, LSPRs can be easily tuned by engineering the composition, size, shape, and local dielectric environment of the nanostructure (Liang et al., 2014).

The past decade has witnessed a significant rise in LSPR enhancement of visible OLEDs with noble metal (Au or Ag) nanostructures resulting in 20–60% improvement in efficiency (Deng et al., 2019; Wu et al., 2018; Munkhbat et al., 2016). Noble metal nanostructures are promising thanks to their unique physical and chemical properties, efficient dispersibility in solvents for solution processing, and moderate costs of production. Chen et al. reported the case of a nearly 100% efficiency enhancement of perovskite LEDs by incorporating Au nanoparticles (NPs) into the hole injection layer of poly(3,4-ethylenedioxythiophene):poly(styrenesulfonate) (PEDOT:PSS) (Chen et al., 2017b). When doped at 9 vol% content in PEDOT:PSS, Au NPs with ∼20 nm diameter feature strong resonant coupling between the LSPR band and the radiated light. Adopting this enhancement strategy could potentially lead to higher efficiencies also in non-toxic NIR-emitting perovskites LEDs, generally characterized by lower efficiencies (see Section 4.1). Recently, a large number of experimental and theoretical works have reported a number of plasmonic nanostructures (e.g., nanospheres, nanorods [NRs], nanoplates, nanocube, nanopores, and core-shell NPs) in which the LSPR can be shifted to the NIR region, shown in Table 2, thus opening to potential coupling with NIR-emitting LEDs. Specifically, Park et al. reported physically synthesized porous Au NPs (PGNs) of size suitable to NIR for biological applications, as shown in Figure 5B, the nanopores in the PGNs shifted the plasmon resonance to the NIR region (Park et al., 2016). The calculated spectra show a red-shifted extinction peak from 650 nm of the non-porous NPs to 800–830 nm of the PGNs. Crucially, the simulation results were well-correlated with the measured extinction coefficients.

**Table 2:** Summary of the shapes, size, resonance absorption peak, and resonance window for plasmonic nanostructures.

Shape	Materials	Size			Resonance peak [nm]^a^	Resonance window [nm]	Ref.
		Diameter/Length [nm]	Width [nm]	Shell [nm]			
Sphere	CS-AuNR-ICG nanosphere	180			720, 805	650–900	(Chen et al., 2013)
	Dpa-Melanin nanospheres	70			700	300–1000	(Liu et al., 2013)
Rod	Au nanorods	50	13		788	650–950	(Zheng et al., 2014)
	Au nanorods	72	16		835	700–1100	(Pan et al., 2019)
	Ag nanorods	142	49.5		790	650–900	(Pietrobon et al., 2009)
Plate	Ag nanoplates	77			700	400–900	(Khan et al., 2017)
	Mg nanoplates	162			700	200–1000	(Biggins et al., 2018)
Cube	Ag nanocubes	80	80		810	450–1200	(Kawawaki et al., 2015)
Pore	Nano-porous Au particles	50			805	550–1100	(Park et al., 2016)
Core-shell	DOX-Au nanosphere	60		10	800	600–1000	(Zhou et al., 2015)
	Ag nanocubes	75		3	830	700–900	(Akselrod et al., 2015)
	Au@Ag/Au nanospheres	7.8	33.0	40.1	900	400–1100	(Ye et al., 2014)
	SiO_2_@Au nanorods	89	16	68	950	600–1100	(Luo et al., 2016)
Other	MoO_2_ irregular nanospheres	70			815	600–1400	(Park et al., 2016)

a Wavelength peak within the 700 to 900 nm spectral window.

Promising as these results might appear, care should be taken however in their use in “*in vivo*” applications. There is, unfortunately, significant concern regarding toxicity and environmental impact of metal nanostructures which, in general, hinders their use in bioapplications (Wijnhoven et al., 2009). For example, Ag NPs show toxicity to biological systems by direct contact with cells and/or release of cytotoxic Ag^+^ ions from its surface (Navarro et al., 2008). Controlling and mitigating the toxicity of metal nanostructures would create new exciting opportunities in the NIR-guided biological field (Sotiriou et al., 2010).

A possible strategy to suppress the toxicity of metal nanostructures is to encapsulate them hermetically within a non-toxic amorphous silica (SiO_2_) shell (Wang et al., 2011). As shown in Figure 5C, NIR resonances can be achieved by switching to aspect ratio (namely L/W is defined as the length/width of the nanostructures) of the SiO_2_-coated Au NRs (Luo et al., 2016). When the aspect ratio increases from 3 to 7.5, the NIR absorption band was observed to shift from 700 to 1150 nm. Notably, silica coating also allows one to adjust the nominal distance between the excitons in the emitting layer with the metal nanostructures ensuring an optimal plasmon-exciton coupling (Munkhbat et al., 2016).

Moreover, non-toxic plasmonic materials are surfacing as alternative solutions to noble metal nanostructures. Dou et al. proposed oxide NCs as a promising NIR plasmon resonance for efficient biocompatible photothermal cancer therapy (Park et al., 2016). Monoclinic MoO_2_ NCs with high crystallinity were successfully synthesized through the combination of laser ablation in liquid and solvothermal synthesis. The MoO_2_ NCs showed intensive LSPR absorption at 600–1400 nm owing to their metallic electronic structure and oxide dielectric function (Figure 5D). Simultaneously, there is no significant cytotoxicity observed for the MoO_2_ irregular nanosphere solution, indicating good biocompatibility.

Incidentally, we also note that Mg NPs can also produce good plasmonic effects (Sterl et al., 2015; Jeong et al., 2016). Recently Biggins et al. revealed multiple size-dependent NIR resonances spanning the entire UV-vis-NIR spectrum (i.e., 440–1000 nm) by combining electron energy loss spectroscopy experiments and simulations to characterize the plasmon modes of Mg nanoplates (Biggins et al., 2018). These Mg NPs are protected by a self-limiting native oxide layer, which renders them stable for several weeks in suspension. Although their toxicity has not been tested yet, earth-abundant Mg might hold great potential to create large numbers of stable and inexpensive plasmonic (NIR) NPs, as also suggested by the authors of that work.

## 4 State-of-the-art NIR-emitting materials

**Figure 6:**
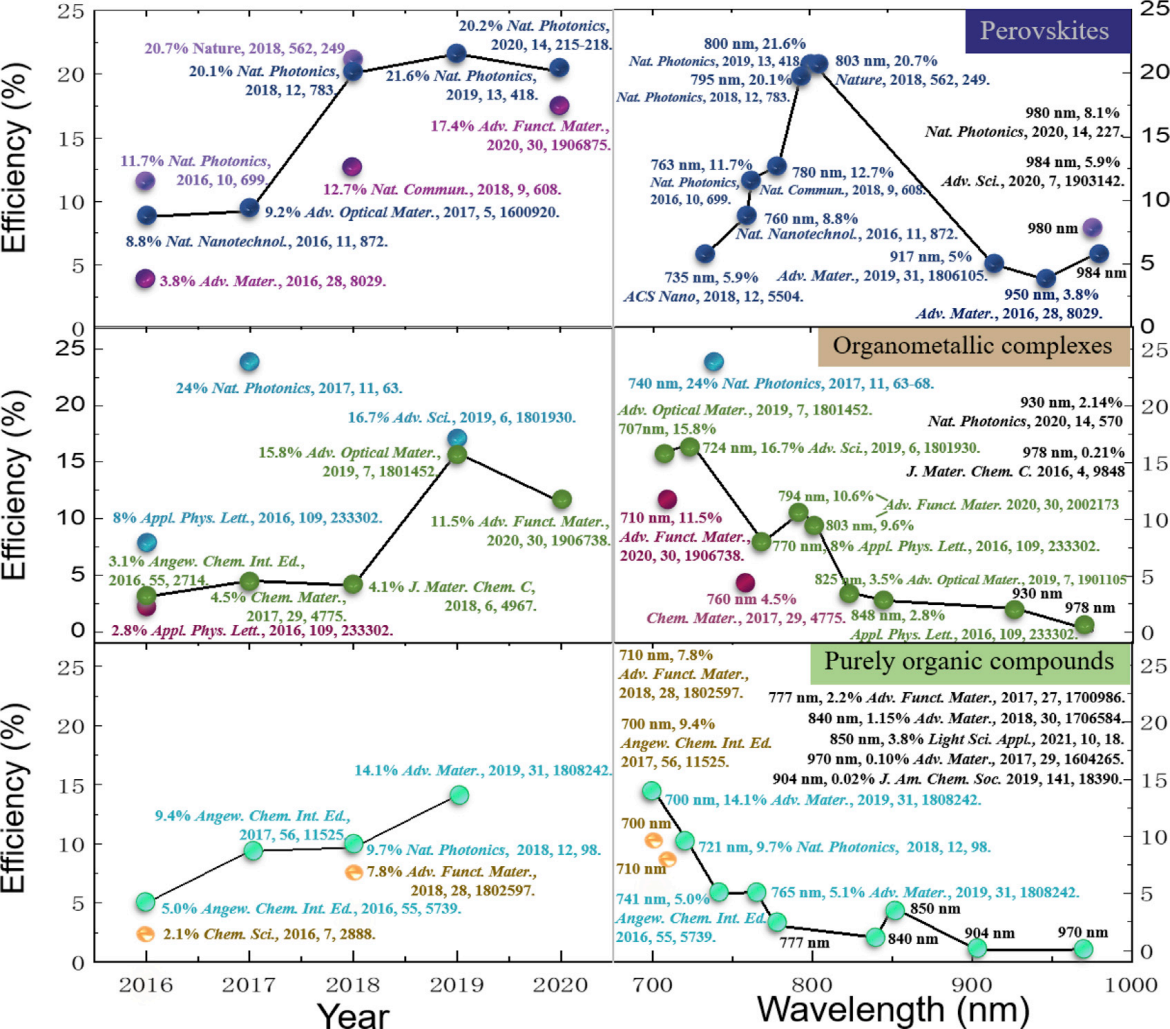
Evolution of state-of-the-art NIR LED efficiencies over the last five years (updated up to 08 November, 2020) Tends of luminescent-material-based NIR LED efficiencies versus publication years (left), and EL peak wavelength (right).

The library of luminescent materials for NIR emission comprises (metal-free) organic semiconductors, metal complexes, and hybrid and inorganic semiconductors. These luminescent materials can provide a flexible molecular design, good electrochemical stabilities, and well-controlled photophysical characteristics. As shown in Figure 6 and Table 3, the rapid growth of state-of-the-art NIR LED efficiencies incorporating different emissive materials over the last five years epitomizes the increasing attention on NIR emitters.

**Table 3:** NIR PL peak wavelength, quantum yield (*Փ*_PL_), NIR EL peak wavelength, and maximum EQE for current state-of-the-art organic-inorganic hybrid and purely organic materials.

Class	Molecule	λ_PL_ [nm]	*Փ*_PL_ [%]	λ_EL_ [nm]	EQE_max_ [%]	Ref.
Perovskite	CsSnI_3_	950	NA	950	3.8^a)^	(Hong et al., 2016)
	PEA_2_(MA)_4_Pb_5_I_16_	760	10.6	760	8.8^a)^	(Yuan et al., 2016)
	NFPI_6_B	NA	67	763	11.7^a)^	(Wang et al., 2016)
	Cs_10_(MA_0.17_FA_0.83_) _(100- x)_Pb(Br_x_I_1-x_)_3_	750	NA	750	9.2^a)^	(Kim et al., 2017)
	NFPI_7_ (2:1.9:2)	780	100^c)^	780	12.7^a)^	(Zou et al., 2018)
	Perovskite-polymer bulk heterostructure	795	96	795	20.1^a)^	(Zhao et al., 2018a)
	FAPbI_3_	800	70	803	20.7^a)^	(Cao et al., 2018)
	EDEA-treated FAPbI_3_	800	56	800	21.6^a)^	(Xu et al., 2019b)
	PbS QD-in-layered perovskite^d)^	935	37	981	8.1^a)^	(Gao et al., 2020)
Phosphorescence	Ir(iqbt)_2_(dpm)	710	16	714	3.1^a)^	(Kesarkar et al., 2016)
	PtNTBP	844	22	848	2.8^b)^	(Huang et al., 2016)
	PtTPTBP	770	51	770	8.0^b)^	(Huang et al., 2016)
	fac-Ir(Ftbpa)_3_	765	14.7	760	4.5^b)^	(Xue et al., 2017)
	Pt(fprpz)_2_	740	81	740	24.0^b)^	(Ly et al., 2017)
	CLZA:0.08Cr^3+^	813	NA	813	4.1^b)^	(Zhang et al., 2018)
	Pt-X-1	660	67.6	707	15.8^b)^	(Cheng et al., 2019)
	F-Pt	727	74	724	16.7^b)^	(Yang et al., 2019)
	Os(ftrmpz)_2_(PPhMe_2_)_2_	737	40	710	11.5^b)^	(Yuan et al., 2020)
	Pt(II) derivative-4^*t*^Bu	960	5.0	930	2.0^b)^	(Wei et al., 2020)
TADF	POZ-DBPHZ	595	79	741 (exciplex)	5.0^b)^	(Data et al., 2016)
	TPA-QCN	700	47	700	9.4^b)^	(Li et al., 2017)
	TPA-QCN (NA film)	733	21	728	3.9^b)^	(Li et al., 2017)
	APDC-DTPA (NA film)	756	17	777	2.2^b)^	(Yuan et al., 2017)
	APDC-DTPA	710	56	710	7.8^b)^	(Hu et al., 2018b)
	Curcuminoid derivative 1	760	45.2	758	5.1^a)^	(Ye et al., 2018)
	Curcuminoid derivative 2	721	70	721	9.7^a)^	(Kim et al., 2018)
	TPAAP	705	69.5	700	14.1^b)^	(Xue et al., 2019)
	TPAAP (NA film)	777	20.3	765	5.1^b)^	(Xue et al., 2019)
	CAT-1	887	0.18	904	0.02^b)^	(Congrave et al., 2019)
Fluorescence	P2TTPD-0.5	667, 874 (shoulder)	6	880	0.15^a)^	(Murto et al., 2016)
	NSeD	670	52	700	2.1^b)^	(Xue et al., 2016)
	BTT^∗^	830	17	840	1.15^a)^	(Minotto et al., 2018)
	TBtz1	661	10	702	1.52^a)^	(Sudyoadsuk et al., 2020)
	TBtz2	686	5	723	1.22^a)^	(Sudyoadsuk et al., 2020)
	eDPP	670, 740 (shoulder)	31	670, 740 (shoulder)	2.72^a)^	(Minotto et al., 2020)

The devices were fabricated by ^a)^ solution-process; ^b)^ thermal evaporation. ^c)^ Film under pulsed laser excitation of 18 nJ cm^−2^. ^d)^ Here perovskites are providing coupling between the QDs and that QDs are the emitters. NFPI_6_B = Ruddlesden-Popper film from NMAI: FABr: PbI_2_ precursors with a molar ratio of 2:1:2, NFPI_7_ = Ruddlesden-Popper film from NMAI: FAI: PbI_2_ precursors with a molar ratio of 2:1:2, EDEA = 2,2′-(ethylenedioxy)-bis-(ethylamine), dpm = 2,2,6,6-tetramethyl-3,5-heptanedionate, iqbt = 1-(benzo[b]thiophen-2-yl)-isoquinolinate, Pt(NTPB) = Platinum (II) aza-triphenyltetrabenzoporphyrin, PtTPTBP = platinum (II) tetraphenyltetrabenzoporphyrin, fac-Ir(Ftbpa)_3_ = facial-tris[1-(2,4-bis(trifluoromethyl)phenyl)-4-(thiophen-2-yl)benzo[g]phthalazine] iridium(III), fprpz = 2-(4-tert-butyl-pyridyl) pyrazole), CLZA = Ca_2_LuZr_2_Al_3_O_12_ garnet, ftrmpz = 2-methyl-5-(3-(trifluoromethyl)-1H-1,2,4-triazol-5-yl)pyrazine, PPhMe_2_ = Dimethylphenylphosphine, POZ-DBPHZ = 3,11-di(10H-phenoxazin-10-yl)dibenzo[a,j]phenazine, APDC-TPA = 3,4-bis(4-(diphenylamino)phenyl)acenaphtho[1,2-b]pyrazine-8,9-dicarbonitrile, TPA-QCN = triphenylamine-quinoxaline-6,7-dicarbonitrile, eDPP = π-expanded diketopyrrolo-pyrrole dye, BTT∗ = triazolobenzothiadiazole, APDC-TPA = 3,4-bis(4-(diphenylamino)phenyl)acenaphtho[1,2-b]pyrazine-8,9-dicarbonitrile, TBtz1-2 see Figure 2A, CAT-1 = see Figure 8D, P2TTPD = poly[3,3′-ditetradecyl- 2,2′-bithiophene-5,5′-diyl-alt-5-(2-ethylhexyl)-4H-thieno[3,4-c]pyrrole −4,6(5H)-dione-1,3- diyl], NSeD = (4,9-bis(4-(2,2-diphenylvinyl)phenyl)-naphtho[2,3-c][1,2,5]selenadiazole

By looking at Table 3, we note that significant progress has been made in the 700 to 800 nm range, with remarkable results in term of EQEs for all the three main classes of NIR LEDs: perovskites, organometallic complexes, and purely organic molecules.

Notably, organic-inorganic metal-halide perovskites, originally popular in photovoltaics, have been successfully applied and intensively studied in LEDs owing to their solution processability and excellent optoelectronic properties. Recent reports by the groups of Huang, Wei, Friend, and Tan have independently demonstrated perovskite LEDs with impressive EQEs greater than 20%, and emission peaks at ∼ 800 nm (Lin et al., 2018; Cao et al., 2018; Zhao et al., 2018a; Zhao and Tan, 2020). However, the inherent toxicity and limited stability of Pb-based perovskite materials have raised considerable concerns. Therefore, finding alternative lead-free perovskite structures, so as to allow preparation of environmentally friendly materials is highly desirable.

Another approach to achieve NIR emission is the use of organometallic complex as phosphorescent emitters, which are able to access triplet states via intersystem crossing from the singlet excited state, thanks to strong spin-orbit coupling mediated by the transition metal (e.g., Ir and Pt) in the complexes. Obviously, the presence of heavy metals again raises concerns for toxicity in these materials.

Alternatively, lanthanide (e.g., Yb, Er, and so on) complexes (often “sensitized” via triplet states) are also of considerable interest for NIR OLEDs because their emission originates from the electronic transitions of the central ions that give sharp narrow spectral characteristic and potentially high emission efficiency. The most significant achievements are reported in Table 3. However, the scarcity of strategic heavy-metal and lanthanide precursor salts is a major setback, potentially derailing their commercial aspirations. Moreover, potential environmental contamination of the heavy metals motivates again a substantial concern.

To overcome these drawbacks, metal-free TADF and traditional “purely fluorescent” materials emerged as potential candidates in tackling the ample range of challenges (e.g., non-toxicity, efficiency, bandwidth, and so on) (Yu et al., 2021). Similar to phosphorescent organometallic emitters, TADF emitters can theoretically harvest all singlet and triplet excitons for light emission and hence theoretically achieve 100% internal quantum efficiencies (Guo et al., 2017; Kaji et al., 2015). This perspective creates further excitements, and we envision future research ahead about TADF emitters in the yet-less-explored NIR region. As shown in the bottom plot of Figure 6, a gradually increasing trend of metal-free LED efficiencies in recent years is observed.

It is important to note that the PLQYs of most fluorescent materials usually become very low in solid films or aggregate state, and their excitons tend to be wasted in nonradiative transition channel. An interesting approach is to exploit the “AIE” molecules featuring propeller-shaped molecular structure, which can form highly emissive aggregates by limiting nonradiative deactivation pathways of the excitations.

### 4.1 Non-toxic/low-toxicity perovskites

The performances of LHPs optoelectronic devices have risen steeply in the last decade. Currently reported efficiencies threaten to challenge the long-established supremacy of traditional inorganic semiconductors in photovoltaics and provide an excellent alternative to organic devices in the field of LEDs (Nayak et al., 2019; Sum et al., 2020). Nonetheless, behind the collective excitement for these results, a widespread concern on LHPs toxicity has been growing within the scientific community. Among other mitigation strategies, coupling LHPs with polymers has also been widely explored to diminish their lead weight content. However, in these devices, the toxicity of lead is exacerbated by its extreme bioavailability (Li et al., 2020; Privitera et al., 2017; Zhao et al., 2018a; Righetto et al., 2020a). As recently demonstrated by Abate's group for LHPs solar cells, setting a threshold for toxic metal content is dangerous because used lead halides salts present a ten-fold higher bioavailability, with respect to other common lead contaminants (Li et al., 2020). Similar considerations can be easily applied to LEDs, which in Europe are tightly regulated under the RoHS regulation (0.1% amount in lead contained) (Babayigit et al., 2016; Conings et al., 2019). Over the past five years, the field of perovskites shifted rapidly to the quest for hybrid or fully inorganic lead-free halide perovskites compounds, capable of achieving comparable performance parameters (Noel et al., 2014).

**Figure 7:**
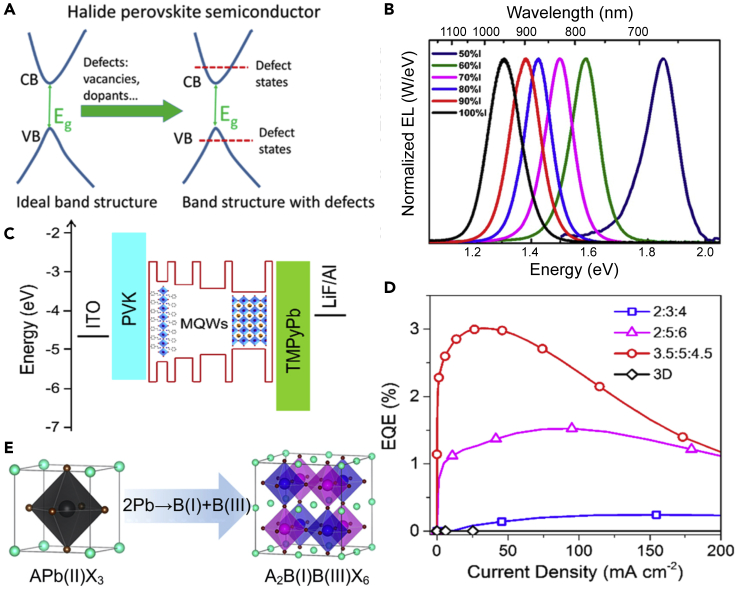
Lead-free halide perovskites for NIR emission (A) Schematic of the defect tolerant electronic structure in halide perovskites. Reproduced and adapted with permission from J. Phys. Chem. C 2018, 122, 46, 26,353–26,361. Copyright 2018, American Chemical Society (Kontos et al., 2018). (B) Tunable electroluminescence from MASn(Br/I)_3_ thin films. Redshift of the EL peak is achieved by increasing the relative concentration of iodide anions. Reproduced and adapted with permission from J. Phys. Chem. Lett. 2016, 7, 14, 2653–2658. Copyright 2016, American Chemical Society (Lai et al., 2016). (C and D) Schematic and EQE curves from NIR emitting quasi 2D tin-based perovskites. Reproduced and adapted with permission from J. Phys. Chem. Lett. 2019, 10, 3, 453–459. Copyright 2019, American Chemical Society (Wang et al., 2019d). (E) Schematic for double perovskites, where lead cations are replaced from isovalent pairs (mono and trivalent cations). Reproduced and adapted with permission from Joule 2018, 2, 1662–1673. Copyright 2018, Elsevier (Zhao et al., 2018b).

Unfortunately, achieving performances comparable with the staggering ones of LHPs-based LEDs (PeLEDs) (Figure 7A) is a tough hurdle to overcome for this novel research field. Among other advantages, low-defect densities, narrow emission peaks, and the balanced charge transport have allowed the fabrication of devices achieving extremely high efficiencies (up to 21.6% EQE_max_ with emissions peaked at 800 nm and high radiance 308 W sr^−1^ m^−2^ at 3.3 V) (Sum et al., 2020; Stranks et al., 2019; Xu et al., 2019b). Such excellent performance parameters mainly originate from a highly dimensional (i.e., with excellent connectivity of frontier atomic orbitals in all three dimensions) (Xiao et al., 2017) and defect-tolerant electronic structure (Figure 7A). Furthermore, their easy solution processing allows inducing partial quantum confinement by means of additives and ligands, which increases exciton binding energies and hence boosts radiative rates (Zhao et al., 2018b; Sum et al., 2020; Park et al., 2019; Righetto et al., 2020a).

As these properties are related to the electronic band structure originating within the octahedral building unit [PbX_6_]^4-^, the substitution of Pb^2+^ cations is a crucial and difficult step (Manser et al., 2016). Initially, the choice was to use other divalent cations from the same group (i.e., Sn^2+^ and Ge^2+^), presenting an analogous lone pair *n*s^2^ *n*p^0^ electronic configuration (Zhao et al., 2018b). Although these cations form analogous perovskite structures, the resulting materials are still plagued by limited chemical stability owing to easier oxidation with respect to lead perovskites. However, encouraging results on stabilizing tin- and germanium-based perovskites pave their way to future research (Chen et al., 2019b). Furthermore, as pointed out by Li et al., their easier oxidation (i.e., the formation of insoluble oxidized products) has a silver lining in terms of environmental impact, as it concomitantly and substantially reduces their bioavailability (Li et al., 2020).

Insofar, tin-based perovskite showed the best performances for NIR light emission. In 2018, Kanatzidis et al. reported intense and highly temperature-stable NIR emission in the range 900–1000 nm for hybrid and fully inorganic ASnI_3_ systems (with A being methylammonium [MA], formamidium [FA], and cesium [Cs] cations [Kontos et al., 2018]). Here, the lower exciton binding energies (e.g., around 12 meV for CsSnI_3_) and higher electronegativity induced by the Sn^2+^ cation make this ideal for NIR emission, causing a considerable redshift with respect to common LHPs (Huang and Lambrecht, 2013). However, as demonstrated by Milot et al., low formation energies for Sn vacancies result in a substantial p-doping, which potentially impairs radiative efficiencies (Yan et al., 2020; Milot et al., 2018).

Tan's group was among the first to demonstrate a MASnX_3_ PeLED (Figure 7B), with 0.72% EQE_max_ at 945 nm (MASnI_3_) and at a radiance of at a radiance of 1.8 W sr^−1^ m^−2^. Moreover, tunable NIR electroluminescence from 700 to 1000 nm was also achieved by introducing bromide anions (Lai et al., 2016). The record EQE for tin-based NIR PeLEDs is currently being held by the fully inorganic CsSnI_3_. In 2016, Hong et al. demonstrated efficient EL from ITO/PEDOT:PSS/CsSnI_3_/PBD/LiF/Al architectures. The outstanding 3.8% EQE_max_ efficiency peaked at 950 nm is still unmatched, albeit plagued by poor stability (Hong et al., 2016). Here, heating-induced degradation concurs with other chemical and electrochemical degradation processes to limit the operational lifetime (L_50_) tin-based PeLEDs to a maximum of 2 h (Dong et al., 2020).

Similar to the case of LHPs, some degree of carrier localization is highly beneficial for boosting emission efficiency in quasi-2D structures, where carriers can funnel and concentrate in the emitting phase (Giovanni et al., 2019). Recently, Wang et al. demonstrated extremely near unity PLQY for visible and NIR-emitting 2D tin-based perovskites (octylammonium)_2_Sn(Br/I)_4_ (Wang et al., 2019a). By using mixed Cs/FA/phenylethylammonium tin iodide perovskites, Wang et al. reported the formation of a quasi -2D tin perovskite (Figure 7C) thin film with emission peaking at 900 nm. Notably, the authors reported a maximum EQE of ∼3%, using polyvinyl carbazole and 1,3,5-tri(m-pyrid-3-yl-phenyl)benzene as transport layers (Figure 7D) (Wang et al., 2019d). Unfortunately, the reported T_50_ ∼ 10 h is far from acceptable in view of commercial applications.

Earlier this year, Liang et al. managed to extend the stability of the red PeLED based on 2D tin perovskite, using ITO/PEDOT:PSS/2D-Sn-perovskite/TBPi/LiF/Al. The resulting highly stable EQE 0.3% is an encouraging milestone, and we envision future work to focus on increasing these efficiencies and shifting emissions of 2D tin perovskites deeper into the NIR (Liang et al., 2020).

Recently, the use of double perovskites has provided a viable alternative to these issues. Introducing heterovalent cations usually forms defect intolerant non-perovskite and A_3_B_2_X_9_ structures with low electronic dimensionality. These properties underlie their relatively poor performances in NIR LEDs, reported by Singh et al (Singh et al., 2019). Here, the authors reported 10^−8^ maximum EQEs peaked at around 800 nm, using Cs_3_Sb_2_I_9_ thin films and conventional PEDOT:PSS and TBPi as transport layers.

Conversely, the combined use of M^+^ (e.g., Ag^+^, Na^+^,K^+^,Rb^+^, and so on) and M^3+^ (e.g., Bi^3+^, Sb^3+^, In^3+^,Tl^3+^, and so on) cations can provide isovalent cation pairs, able to replace the lead within perovskite structures (Zhao et al., 2018a). Resulting double perovskites or “elpasolites” present the structure A_2_ M(I)M(III)X_6_ and can be categorized into different types based on the constituent M cations (Figure 7E). For a comprehensive review on lead-free double perovskites, we refer the reader to the excellent work by Zhao et al (Zhao et al., 2018a). Albeit stable and relatively non-toxic, not all double perovskites are direct bandgap semiconductors, thus limiting their use in LEDs (Khalfin and Bekenstein, 2019). While only visible and white emitting LEDs were reported for double perovskites, we envision their possible use as doping host for "quantum cutting" applications. As demonstrated by Gamelin et al, LHP hosts doped with ytterbium ions can reach staggering 170% PLQYs in the NIR (Milstein et al., 2018). The underlying quantum cutting has been applied to lead-containing NIR LEDs by Tom Miyasaka, reaching an outstanding 5.9% EQE at 984 nm (Ishii and Miyasaka, 2020; Righetto et al., 2020a). Future work will certainly focus on reproducing similar results with lead-free perovskites.

The field of NIR-emitting lead-free PeLEDs is still at early development. However, the promise of high efficiencies and facile fabrication will continue to attract interest, paving the way for future developments in this field.

### 4.2 Leveraging triplet to singlet conversion: triplet-triplet annihilation versus TADF

As mentioned previously, organic TADF emitters can potentially exploit both singlet and triplet states, generated upon electrical injection in OLEDs (i.e., increasing the apparent *r*_*st*_ factor) for luminescence (Liu et al., 2018b). The possibility of harnessing the triplet excitons' contribution without rare and expensive heavy metals arises from a photophysical process converting otherwise “dark” triplets into emissive singlets (Kaji et al., 2015). Generally, this triplet-to-singlet interconversion process can follow two distinct mechanisms, namely triplet-triplet annihilation (TTA) or TADF sometimes also indicated as reverse ISC (rISC). In the TTA mechanism, the yield of singlet emissive states is highly dependent on the relative energy order of the excited singlet and triplet energy levels in the molecule, and the maximum total singlet yield is in principle limited to 62.5% (Kondakov et al., 2009) or lower if considering more sophisticated photophysics (Monkman, 2013). In practice, TTA only becomes efficient at high densities of triplets (Kondakov, 2015), and no efficient TTA-based NIR LEDs have been reported insofar, to the best of our knowledge.

TADF/rISC occurs by thermal activation of triplet excitons to the singlet manifolds and therefore requires the energy difference between singlet and triplet energies (Δ*E*_ST_) to be as small as possible (Uoyama et al., 2012). Notably, exploiting all the triplet excitons can in principle lead to a nearly unitary internal quantum efficiency (provided *η*_*PL*_ is ≈ 1) (Chen et al., 2019a; Kaji et al., 2015).

**Figure 8:**
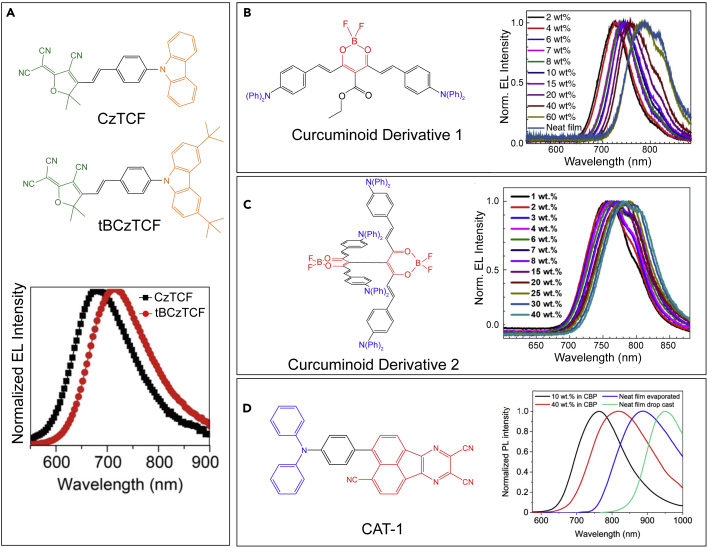
Examples of NIR-emitting TADF molecules (A) Chemical structure and normalized EL spectra of CzTCF and tBCzTCF (Zhao et al., 2019). (B and C) Left: Chemical structure of the curcuminoid derivation 1 and 2. Right: NIR EL spectra for various doping concentrations of curcuminoid derivations. Reproduced and adapted with permission from Nat. Photon. 12, 98–104 (2018). Copyright 2018, Springer Nature. Reproduced and adapted with permission from Chem. Mater. 2018, 30, 19, 6702–6710. Copyright 2018, American Chemical Society (Kim et al., 2018; Ye et al., 2018). (D) Molecular structure of CAT-1 and normalized steady state PL spectra for CAT-1 in doped and neat films. Reproduced and adapted with permission from J. Am. Chem. Soc. 2019, 141, 46, 18,390–18,394. Copyright 2019, American Chemical Society (Congrave et al., 2019).

The adequate separation of spatial distribution for the HOMO and LUMO is a viable strategy to minimize the Δ*E*_ST_ values of conjugated molecules. A general approach is to design twisted D-A-conjugated oligomers with a significant intramolecular charge-transfer (ICT) character, which has been applied to the vast majority of NIR TADF candidates published insofar (Wong and Zysman-Colman, 2017). For example, Li et al. synthesized two similar D-*π*-A TADF compounds (CzTCF and tBCzTCF) by using 2-dicyanomethylene-3-cyano-4,5,5-trimethyl-2,5-dihydrofurance (TCF) with strong electron-withdrawing ability as acceptor and corresponding carbazole (Cz) and 3,6-di-*tert*-butyl-9*H*-carbazole(tBCz) as donor units (Zhao et al., 2019), as shown in Figure 8A. In the non-doped LEDs, far-red/NIR emissions peaked at 683 and 715 nm is achieved for CzTCF and tBCzTCF, respectively.

It is well known that the ICT effects can induce a remarkable bathochromic shift of the emission, especially for the molecules with extended conjugated frameworks. In 2018, Adachi et al. reported a donor-acceptor-donor-type TADF curcuminoid derivative consisting of one acetylacetonate boron difluoride acceptor and two triphenylamine units (Kim et al., 2018). By blending this compound with CBP host, they achieved EQEs approaching 10% with EL peaking at 721 nm, also the EL peak wavelength can be tuned from 700 to 780 nm via controlling concentration of curcuminoid derivative (shown in Figure 8B). Such performances exceed those reported so far in conventional NIR fluorescent emitter-based devices, presenting an important advance in NIR OLEDs.

Alternatively, there has been great interest toward acceptor cores functionalized with multiple peripheral donors, that is, polydonor D_*n*_−A structures where *n* > 1 (Wong and Zysman-Colman, 2017; Zampetti et al., 2019; Congrave et al., 2019). To shift the EL further toward the NIR, Adachi et al. used such a scheme to the curcuminoid derivative and demonstrated another D_4_-A_2_ analog with the same TADF character, leading to an EL wavelength of 758 nm with an EQE of 5.1% (Figure 8C) (Ye et al., 2018). In particular, this molecule emits at λ_max_ = 801 nm with a PLQY of 4% at 40 wt% curcuminoid derivative in CBP host.

Even in the presence of significant progress, as highlighted previously, the increased number of donors actually suppresses the strength of the acceptor, compromising any redshift. For some TADF materials (Tanaka et al., 2013; Zhang et al., 2014; Yu et al., 2018), any redshift in emission afforded by additional donors is incremental at best compared with single D-A dyads. Bronstein et al. recently showed that the use of multiple donors is ineffectual at redshifting the emission. However, the use of simple D-A systems with a sufficiently strong D-A interaction leaves much space for further rational functionalization. The authors exploited that to greatly stabilize the internal charge transfer state and obtained a dramatic redshift (up to 100 nm).The resulting TADF emitter in the evaporated and drop-cast films was capable of PL peaking at 887 and 950 nm, respectively (Figure 8D) (Congrave et al., 2019). In preliminary thermally-evaporated LEDs, the undoped devices exhibit complete NIR emission with an EL peaking at 904 nm and a very low EQE of 0.019%. Although these results confirmed that the long-wavelength PL of the evaporated film of CAT-1 can be retained in an LED device, the solution-processed LED was not presented. It is a common phenomenon because laminating multiple layers by a solution process is still a challenge for TADF OLEDs.

Thanks to the great efforts dedicated to the development of these materials, efficient TADF polymers are recently emerging and have been successfully realized in visible PLEDs (Liu et al., 2020c; Zeng et al., 2018). For example, Wang et al. demonstrated that the non-doped electroluminescent devices with the TADF polymers produce red emission with an EQE_max_ of up to 12.5% and the emission peaked at 620 nm (Wang et al., 2020b), which represents state-of-the-art performance for solution-processed devices based on red TADF polymers. We can envision that such advances in the field of polymeric TADF emitters could be harnessed and translated to the NIR range. Therefore, we believe that future efforts on the TADF polymers for NIR PLEDs will focus in this direction.

### 4.3 AIE fluorophores

For most conventional organic dyes, the contiguity between aromatic rings of neighboring fluorophores often promotes strong π-π stacking interactions, thereby favoring a significant formation of aggregates with random or ordered structures (Hong et al., 2009). The excited states of the resulting aggregates usually decay via non-radiative pathways, thus inducing partial or complete emission quenching. This phenomenon is known as aggregation-caused quenching and has significantly impaired the use of molecules as condensed phase emitters and has led researchers to use dilute solution/doped films for LEDs applications (Mei et al., 2015). This consideration specially applies to extensively conjugated NIR moieties, which exhibit a higher degree of molecular planarity compared with visible emitters. Planarity favors the formation of poorly emissive cofacial (H-type) aggregates and therefore induces a more quenched emission with respect to visible luminophores with a larger gap (Spano and Silva, 2014).

In 2001, Tang et al. reported that a series of silole derivatives showed weak or negligible emission in dilute solutions but became highly luminescent when the molecules were aggregated in concentrated solutions or cast into solid films, by means of an “AIE” process (Luo et al., 2001). Since their pioneering work, a large number of derivatives and analogs functionalized with freely rotating peripheral aromatic rings have been discovered, quickly becoming a hot frontier research topic (Mei et al., 2015; Chen et al., 2019c). In fact, AIE fluorophores have been successfully applied to efficient OLEDs as well as highly selective fluorescence sensors for biological and chemical analytes (Mei et al., 2015). Among the different possible strategies to achieve AIE, steric hindering by means of bulky peripheral groups emerged as the most widely used one. This strategy has a two-fold aim: (i) limiting the π-π molecular stacking responsible for aggregation-induced quenching (i.e., the formation of H-aggregates) and (ii) circumventing non-radiative relaxation pathways (Li and Li, 2017; Zong et al., 2016).

**Figure 9:**
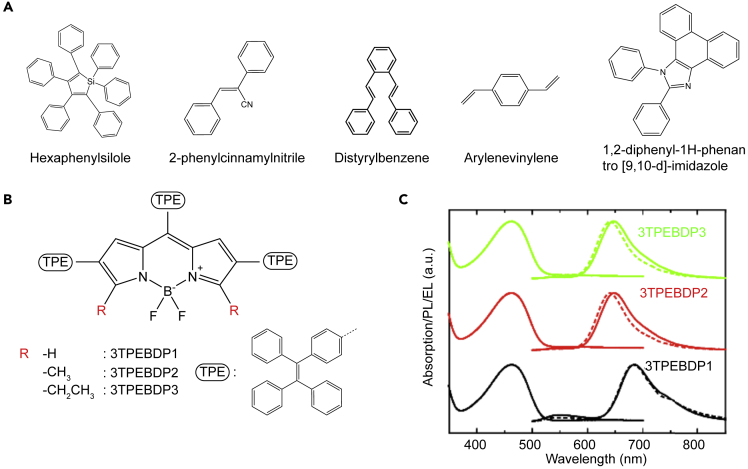
Examples of NIR-emitting BODIPY derivatives displaying aggregation-induced emission (A) Chemical structures of AIE active groups. (B) Molecular structure of 3TPEBDPx. (C) Absorption, PL, and EL spectra (dashed lines) of 3TPEBDPx: F8BT blends with 1 w/w% of 3TPEBDPx loading. Reproduced and adapted with permission from Sci. China Chem. 61, 932–939 (2018). Copyright 2018, Springer (Baysec et al., 2018).

In addition to the aforementioned n-phenyl siloles (Zhao et al., 2015; Zhang et al., 2015), other classes of commonly used AIEgens include tetraphenylethylene (TPE) derivatives (Feng et al., 2018), 2-phenyl cinnamyl nitrile (An et al., 2012; Li and Zheng, 2011), distyrylbenzene (Shi et al., 2012; Gierschner et al., 2013), and arylenevinylene derivatives (Hong et al., 2011), as shown in Figure 9A.

Despite the promising achievements obtained in the visible range, efficient NIR emission from such fluorophores has been rarely demonstrated. To fill this gap, researchers have focused on alternative AIE-active materials, such as 1,2-diphenyl-1H-phenanthro[9,10-d]-imidazole (PPI) (Figure 9A). Early this year, by using PPI as the donor and benzothiadiazole as the acceptor, Liu et al. reported an efficient deep-red/NIR AIE fluorophore with a high PLQY of 35% in neat films (Liu et al., 2020a). The nondoped OLED based on such an emitter achieved a maximum EQE of 2.02% with an EL peak at 672 nm. At the same time, Tang et al. introduced PPI unit as a large planar substitution group to a triphenylamine-modified naphtho[2,3-c][1,2,5]thiadiazole unit to synthesize an AIE-active NIR emitter. One of derivatives exhibited EL emission peaking at 686 nm with an EQE of 2.48% (Wan et al., 2020).

AIE in the same spectral range was also demonstrated in a series of emitters based on 4,4-difluoro-4-bora-3*a*,4*a*-diaza-*s*-indacene (Baysec et al., 2018) decorated with AIE-active TPE groups Figure 9B. Such molecules displayed PLQY reaching up to 50% in the solid state as neat thin films and up to 100% when dispersed in F8BT blends. By incorporating such blends in the active layer of solution-processed PLEDs, showed EL peaked between 650 and (nearly) 700 nm (Figure 9C), with an EQE up to 1.8%. Nevertheless, almost all of low-gap AIE molecules reported so far emit in the far-red spectral region from 650 to 700 nm. This could be attributed to the presence of freely rotating peripheral units in AIE molecular units, which inherently reduce π-electron delocalization lengths and therefore induce a hypsochromic shift of optical transitions with respect to rigid molecules.

Interestingly, a synergy between the AIE phenomenon and the TADF process has recently been proposed to develop novel and robust luminescent materials (Kim et al., 2019; Ma et al., 2019). For example, Tsujimoto et al. reported three 9,9-dimethylxanthene bridged D-A molecules bearing phenothiazine, carbazole, or 3,6-di-*tert*-butylcarbazole as donor groups (Tsujimoto et al., 2017). Crucially, the through-space charge transfer is mediated by spatial π-π interactions because the donor and acceptor groups are placed in close proximity. Such structured molecules exhibited delayed fluorescence in the absence of triplet-quenching oxygen in both solution and solid state, which are characterized as TADF characteristics, and enhanced quantum yields in the solid state. The yellow OLED devices incorporate XPT as the emitter displayed EQE as high as 10%, which further inspired more interesting research toward NIR emission.

### 4.4 Other classes of NIR emitters

The interplay between ionic and electronic charge carriers in mixed conductor materials offers rich physics and unique device potential (Slinker et al., 2007). In light-emitting electrochemical cells (LECs), for instance, the redistribution of ions assists the injection of electronic carriers and leads to efficient light emission. Aiming to shift the EL deeper into the NIR, a novel class of metal-free emitters for LECs was reported. In 2013, Bolink et al. achieved an EQE of 0.44% peaked at 700 nm from a metal-free cyanine-based NIR-LEC driven by a high-frequency pulsed current (Pertegás et al., 2013). Wang et al, in 2017, demonstrated an LEC comprising a metal-free alternating copolymer as the emitter, delivering EL, emission peaked at 705 nm with a high radiance of 129 *μ*W cm^−2^ (Tang et al., 2017). Very recently, Wang et al. also studied a set of host-guest copolymers with alternating benzodithiophene and benzotriazole derivatives as host units and 4,7-bis(5-bromothiophen-2-yl)-benzo[*c*][1,2,5]thiadiazole as the minority guest (Xiong et al., 2019). Such host-guest copolymer emits at λ_max_ = 723 nm with an EQE of 0.135% in LEC device. These examples constitute the current state-of-the-art metal-free active compounds for application to NIR-emitting LECs.

Squaraine derivatives are another promising class of molecules which show excellent photochemical and thermal stability, and also benefit from a narrow absorption band in the visible and in the NIR region (Beverina and Salice, 2010). Owing to their simple synthesis and good environmental stability, squaraine dyes have been successfully used in a number of technologically relevant applications such as dye-sensitized solar cells (Alagumalai et al., 2016), field-effect transistors (Maeda et al., 2018), photovoltaic cells (Chen et al., 2017a), photodetectors (Strassel et al., 2018), nonlinear optics, or bioimaging (Chang et al., 2019). In particular, Stender et al. reported an EL peaked at 550 and 730 nm with EQE up to 0.65% from LEDs incorporating a bromoindolenine squaraine dye into poly(phenylenevinylene) (Stender et al., 2013). Harkin et al. also reported in 2016 a purely NIR LED peaked at 800 nm with EQE of 0.2% by using efficient resonance energy transfer from polymer host poly(indacenodithiophene-*alt*-benzothiadiazole) to squaraine guest ter[bis(indolenine) dicyanomethylensquaraine] (Harkin et al., 2016).

In addition, remarkable progress has been achieved by adopting single-walled carbon nanotubes (SWCNTs) as alternative organic NIR emitters. Matching SWCNT EML with charge-blocking materials and doped charge transport layers, SWCNT-based LEDs exhibited narrow-band EL emission at wavelengths between 1000 and 1200 nm. Despite the EQE of the device being very low (0.014%), the authors demonstrated that the EL emission could be further tuned across the entire NIR range by using SWCNTs with different diameters and chirality or through chemical modification (Graf et al., 2018).

Furthermore, colloidal quantum dots (QDs) are emerging as promising materials for constructing NIR emission in view of their tunable luminescence, high quantum efficiency and compatibility with solution processing. However, these NIR QDs have been raising much concern for the potential release of toxic ions such as Cd^2+^, Hg^2+^, Pb^2+^, and As^3−^, which pose risk to human health and environment under certain conditions (Xu et al., 2016; Sun et al., 2013). To overcome this limitation, biocompatible coating is frequently introduced to realize core/shell nanostructures, thus helping not only to increase the biocompatibility of QDs but also to passivate surface defects. For example, Wijaya et al. demonstrated a NIR LED based on giant shell In(Zn)As-In(Zn)P-GaP-ZnS III-V QDs that exhibited EL emission peaked at 857 nm with an EQE up to 4.6% (Wijaya et al., 2020). Nevertheless, such a device still suffers from the presence of potentially toxic elements (e.g., In, As), which could trigger toxicity concerns. Interestingly, early this year, novel doped I-III-VI QD without lead or other toxic elements were reported, although this has not yet been exploited in LEDs (Du et al., 2020). It is therefore reasonable to expect that toxic-element-free QD will be applied to LED for NIR emission.

## 5 Conclusions and future perspectives

The field of NIR LEDs based on purely organic and hybrid semiconductor materials is attracting exceptional attention, and their performance, particularly in terms of device quantum efficiency, has significantly improved. Noteworthy, a clear focus on solution-processing and toward the exploitation of non-toxic materials is driving this field. Given the potential impact of non-toxic NIR-OLEDs in many fields of application here discussed (e.g., security, communications, wearable biosensors, and photodynamic therapy), we see a clear opportunity for technological transfer to the industry, although transfer from a “laboratory scale” to an “industrial” one will surely need significant translational effort.

From the viewpoint of NIR light generation, efficient photoluminescence needs to be combined with balanced carrier injection/transport, low efficiency roll-off, good color stability, and long operation lifetime. The development of active layers has catalyzed the development observed over the past years, with the overall device architecture remaining essentially unchanged. Host-guest-based active layers have so far afforded state-of-the-art NIR LEDs efficiencies, and we expect that this trend will continue, for example in terms of “hyperfluorescence” strategies, developed by Adachi's laboratory and Kyulux, Inc (Chan et al., 2021; Shuo-Hsien et al., 2018). Clearly, there is still much research to be performed to improve NIR LEDs. We believe sub-wavelength-sized plasmonic nanostructures maintain a largely unexplored potential to further boost the performance of the NIR LEDs.

The most efficient NIR-emitting devices are based on hybrid perovskites with well-defined nanoscale morphology and phosphorescent complexes, reporting efficiencies greater than 20%. Here, heavy metals play a pivotal role in redefining the role of trap states (e.g., in perovskites) and manipulate the spin multiplicity of the excited states (e.g., in heavy metal complexes). Therefore, the formidable challenge in front of the community concerns the attaining of comparable properties in heavy-metal-free materials. The use of isovalent ionic pairs to substitute the Pb(II) in the inorganic case and the engineering of exchange interaction to recover the triplets in the organic case are the two exciting emerging fields. Furthermore, NIR TADF show performance metrics comparable to, and in some cases improved upon, current state-of-the-art organometallic complexes. Nevertheless, realizing high-performance solution-processable TADF devices for NIR, particularly in the long wavelength range (greater than 800 nm), remains a challenge. In addition, organic fluorophores leveraging AIE and other promising classes of emitter materials, including metal-free emitters for LECs, squaraine derivatives, carbon nanotubes, and low-toxicity quantum dots, provide an encouraging playground for further developing of NIR electroluminescence.

Last but not least, to fulfill commercial aspirations, the development of these materials cannot overlook the manufacturing aspect, which however is beyond the scope of this review.

In the future, further attention should be paid to the quality of the NIR light emitted by non-toxic LEDs. Although many devices feature NIR EL, in many cases, the emission spectrum often falls in the deep-red spectral region. The way forward is to ensure that the fraction of photons emitted above 700 nm is 90%, while maintaining high efficiency and long operation lifetime.

Further development of alternative electrode materials replacing the brittle, expensive, and potentially toxic ITO will also play a key role in lowering the costs of deposition processes, thus improving the viability of printing techniques.
